# Essentiality, protein–protein interactions and evolutionary properties are key predictors for identifying cancer-associated genes using machine learning

**DOI:** 10.1038/s41598-023-44118-2

**Published:** 2024-04-22

**Authors:** Amro Safadi, Simon C. Lovell, Andrew J. Doig

**Affiliations:** 1 Division of Evolution and Genomic Sciences, School of Biological Sciences, Faculty of Biology, Medicine and Health, The University of Manchester, Manchester, M13 9PT UK; 2 Division of Neuroscience, School of Biological Sciences, Faculty of Biology, Medicine and Health, The University of Manchester, Manchester, M13 9BL UK

**Keywords:** Cancer genetics, Bioinformatics, Cancer genetics, Cancer genomics, Machine learning, Cancer genetics

## Abstract

The distinctive nature of cancer as a disease prompts an exploration of the special characteristics the genes implicated in cancer exhibit. The identification of cancer-associated genes and their characteristics is crucial to further our understanding of this disease and enhanced likelihood of therapeutic drug targets success. However, the rate at which cancer genes are being identified experimentally is slow. Applying predictive analysis techniques, through the building of accurate machine learning models, is potentially a useful approach in enhancing the identification rate of these genes and their characteristics. Here, we investigated gene essentiality scores and found that they tend to be higher for cancer-associated genes compared to other protein-coding human genes. We built a dataset of extended gene properties linked to essentiality and used it to train a machine-learning model; this model reached 89% accuracy and > 0.85 for the Area Under Curve (AUC). The model showed that essentiality, evolutionary-related properties, and properties arising from protein–protein interaction networks are particularly effective in predicting cancer-associated genes. We were able to use the model to identify potential candidate genes that have not been previously linked to cancer. Prioritising genes that score highly by our methods could aid scientists in their cancer genes research.

## Introduction

The identification of cancer-related genes (referring to both oncogenes and tumor suppressor genes) remains a key challenge. Among all human genes, approximately 3.5% have been directly implicated in cancer initiation and progression^1^, though it is likely that many remain to be found. Accurate identification of genes potentially related to cancer would provide an opportunity to advance both personalised treatment of cancer and aid drug discovery by providing new targets. The Cancer Gene Census of COSMIC^1^ provides an expert-curated dataset of cancer-associated genes, relying on tumor sample analysis to identify cancer genes. This provides a high standard in accurately identifying these genes. However, expert-curation is a lengthy and complex process due to several factors including the availability of tumor samples and the difficulty in sequencing them. Several studies have attempted to build models to identify human disease-related genes. Computational models built using sets of evolutionary and protein network-based properties showed great potential and success in predicting disease genes^2,3^. Using protein–protein interaction properties also showed great potential in cancer gene prediction when compared to the frequency of mutations based approach^4^. However, the goal of accurately predicting cancer genes still eludes us, despite multiple approaches that have been attempted to date.

One viable approach may be to define and enrich the set of properties that characterise cancer genes and combine these properties to reach a more reliable prediction method. Several characteristics may be correlated with the likelihood of a gene being associated with cancer. A prime candidate is essentiality. A gene is considered essential when loss of its function compromises the viability of an individual^5^. Essentiality is a quantitative measure and not a simple divide between essential versus non-essential, as defining it as such would be impossible due to the changeable nature of essentiality based on the genetic and environmental context. The identification of essential genes in multiple organisms has provided researchers with vital insights into the mechanisms of biological processes^6^. For example, essential genes are likely to encode hub proteins in protein–protein interaction networks, signifying more interacting partners than non-essential genes. Furthermore, essential genes are more likely to be abundantly and ubiquitously expressed in cells and tissues and have smaller-sized introns^7^. Also, several studies determined the relationship between evolutionary conservation and the degree of essentiality in genes with variations in findings across species^7^. The general findings in human genes point to a relationship whereby the more essential the gene is, the less likely it is to show enrichment of missense mutations. In contrast, the number of synonymous mutations is not dependent on essentiality. This indicates that purifying selection acts more stringently on essential genes^5,8^.

One could argue that genes implicated in driving and initiating tumors, which generally do not compromise viability in a direct manner, are thus unlikely to score high on the essentiality spectrum. However, there are indications that human genes associated with genetic disease are likely to be essential^6^. Cassa et al.^8^ investigated heterozygous protein-truncating variants in over 60,000 individuals from the Exome Aggregation Consortium (ExAC) dataset^9^ using the ‘s_het_’ essentiality score (a metric that provides Bayesian estimates of the selection coefficient against heterozygous loss-of-function variation) and were able to predict phenotypic severity, age of onset and penetrance for Mendelian disease-associated genes. In addition, genes involved in neurological phenotypes, including autism, congenital heart disease and inherited cancer risk, seem to be under more intense purifying selection, which may indicate essentiality. Overall, quantitative estimates of essentiality appear to be particularly useful in Mendelian disease gene discovery efforts.

Here, we identify combinations of gene properties that have not been previously used to assess the likelihood of a gene to be cancer-associated. We study whether cancer-associated genes are more likely to be essential than non-cancer genes, and check whether an uplift in predicting a gene to be a cancer-related can be achieved by using essentiality-related properties. These findings can also indicate whether these genes are more likely to be under stronger selection than other non-cancer related genes. We were able to build a relatively accurate machine-learning model for predicting cancer genes using essentiality-related properties. Using this machine learning approach, we were able to identify further candidate genes for cancer, in addition to those currently reported in COSMIC census (October 2018).

**Figure 1:**
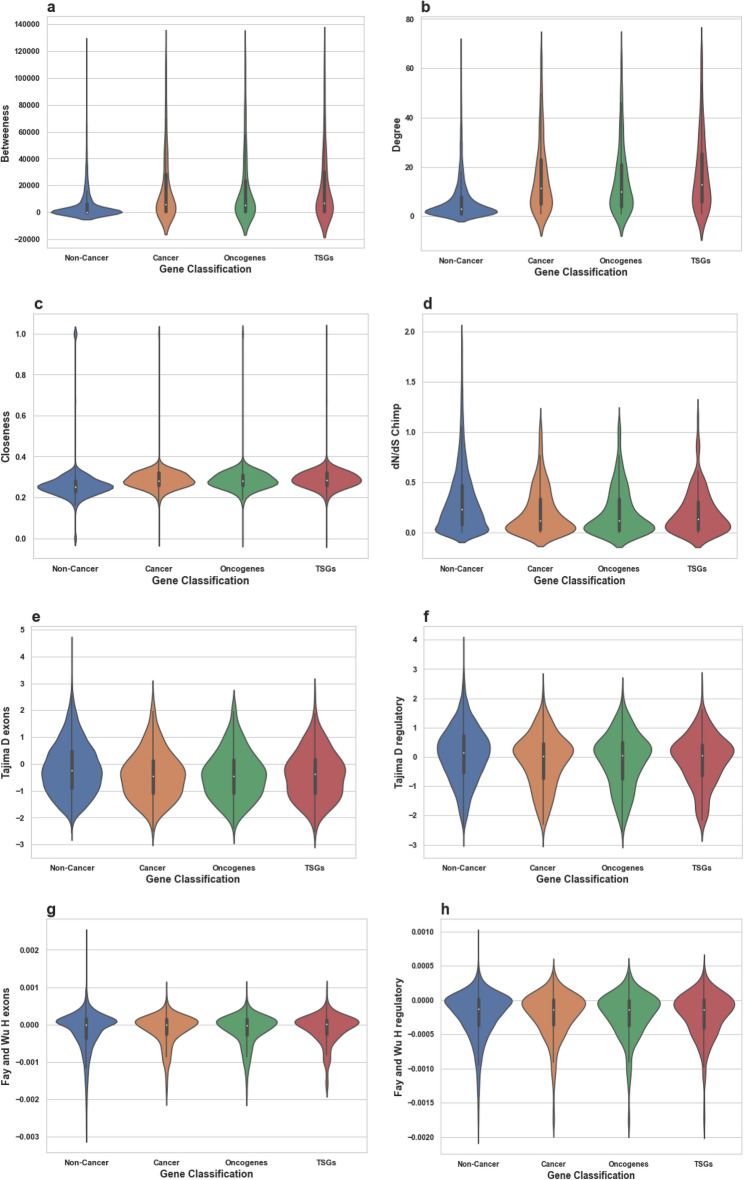

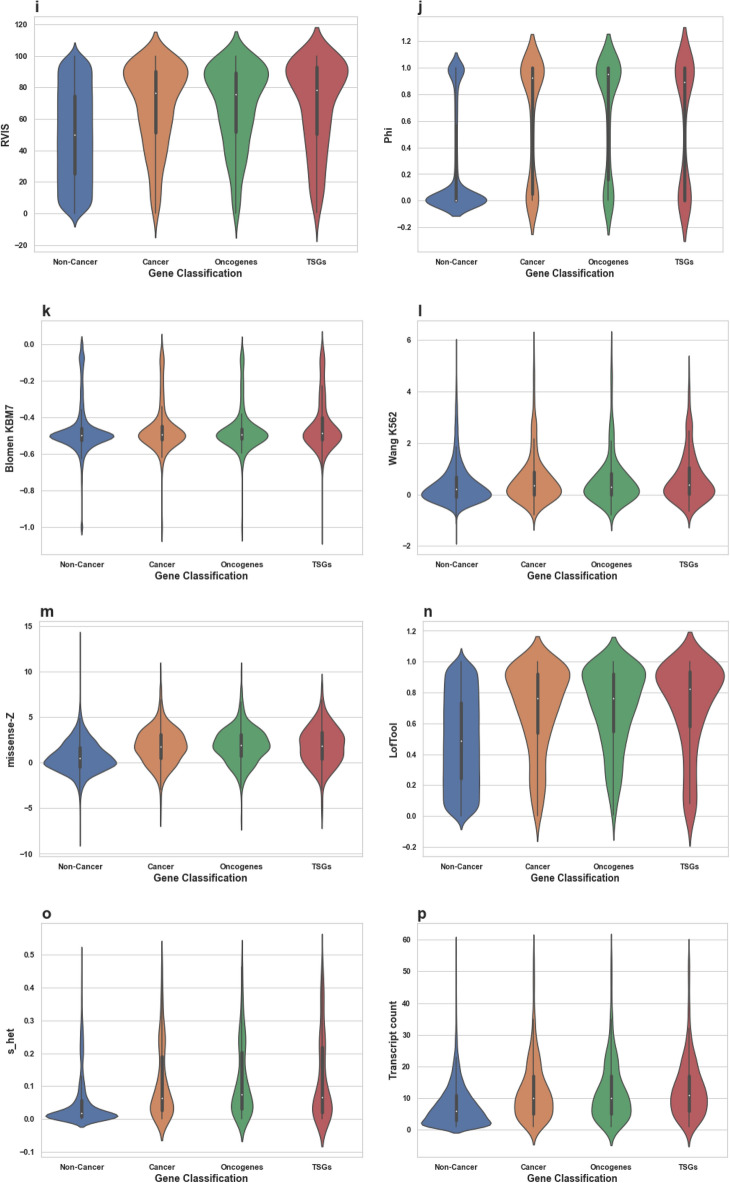

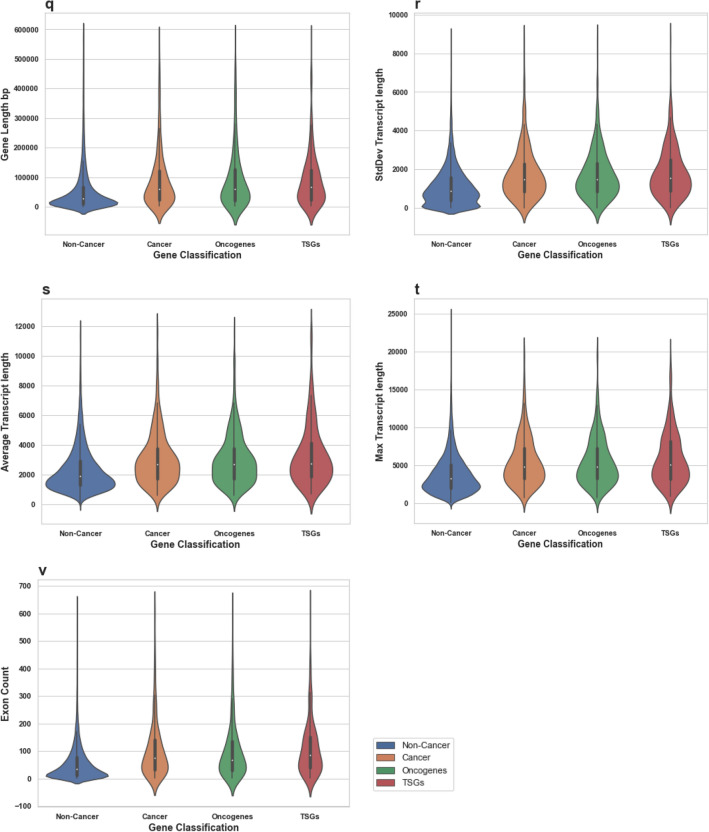
Distributions of all tested features’ values for Non-Cancer and Cancer genes. These violin plots outline distributions of: (**a**) Betweenness (**b**) Degree (**c**) Closeness (**d**) dN/dS Chimp (**e**) Tajima D exons (**f**) Tajima D regulatory (**g**) Fay and Wu H exons (**h**) Fay and Wu H regulatory (**i**) RVIS (**j**) Phi (**k**) Blomen KBM7 (**l**) Wang K562 (**m**) missense-Z (**n**) LofTool (**o**) s_het_ (**p**) Transcript count (**q**) Gene Length bp (**r**) StdDev Transcript length (**s**) Average Transcript length (**t**) Max Transcript length and (**v**) Exon Count with overlaid boxplots. The width of the violin plots represents the proportion of the data located there; the top and bottom of the boxplots denote the upper and lower quartiles; the white dot inside the box denotes the median of the data.

## Materials and methods

A total list of 18,000 human protein-coding genes and their properties was obtained by combining data from different data sources^3,5^. The complete dataset (later used to train a machine learning model) is available in the Supplementary Information Table 1.

**Table 1:** Comparison between the mean essentiality scores of cancer genes, tumour suppressor genes, oncogenes and all other human genes.

Method	Mean essentiality score for all genes	Mean essentiality score for cancer genes	Ratio (cancer genes/all genes)	*P* values	Mean essentiality score for TS genes	Ratio (TS genes/all genes)	*P* values	Mean essentiality score for oncogenes	Ratio (Oncogenes/all genes)	*P* values
Phi	0.27	0.63	2.34	< 0.00001	0.58	2.17	< 0.00001	0.67	2.48	< 0.00001
Wang	0.42	0.62	1.48	< 0.00001	0.65	1.55	< 0.00001	0.57	1.36	< 0.00001
S_het	0.06	0.12	2.07	< 0.00001	0.12	2.07	< 0.00001	0.12	2.04	< 0.00001
LofTool	0.50	0.70	1.40	< 0.00001	0.71	1.42	< 0.00001	0.70	1.40	< 0.00001
Missense-Z	0.69	1.86	2.70	< 0.00001	1.85	2.69	< 0.00001	1.93	2.80	< 0.00001
RVSI	50.0	68.3	1.37	< 0.00001	68.9	1.38	< 0.00001	68.32	1.37	< 0.00001

### Essentiality scores

We obtained several different essentiality scores calculated for human genes from^5^. Petrovski's 'residual variation intolerance score' (RVIS)^10^ and Rackham's EvoTol^11^ relate the amount of common loss-of-function variation to the total gene variation. Other scores are based on the work of Samocha et al. (Missense Z-score)^12^, which sets up a baseline expectation of mutation count per gene based on the sequence context, local mutation rate, sequencing depth and, most importantly, sample size. Fadista's LoFtool^13^ combines the neutral mutation rate of Samocha et al. with the evolutionary information in EvoTol. The baseline neutral expectation is compared with the observed counts of loss-of-function variants in the Missense Z-score, in Bartha's probability of haploinsufficiency (Phi)^14^, and in Lek's probability of loss-of-function intolerance (pLI)^15^. Finally, recent work by Cassa et al.^8^ describes a metric (s_het_) that provides Bayesian estimates of the selection coefficient against heterozygous loss-of-function variation. The various scores were developed or updated using the Exome Aggregation Consortium (ExAC) sample of 60706 human exomes described in^15^. These scores show high correlations to one another^5^.

### Evolutionary profile and genomic related properties

We used gene properties provided and constructed in^3^ including genomic location, protein network parameters and summary statistics of neutrality for human genes. The genomic location properties we used in our work were: Chr, Start, End and Strand and additionally dN/dS values that indicate neutrality and selection pressure (multiple species). All were extracted from Ensembl Biomart Genes^16^.

Group property divides genes into three different mutually excluding groups: (i) Complex-Mendelian (CM) genes, (ii) Mendelian Non-Complex (MNC) genes, and (iii) Complex Non-Mendelian (CNM) genes.

Data also includes measures of genetic variation at intra-species level, and measures for proportions of rare variants, such as Tajima's D and Fay and Wu's H values, for both exons and regulatory regions^3^.

### Protein network properties

The human protein–protein interaction network (PIN) was reconstructed from the interactions available in the BioGRID database version 3.1.81^17^. Properties, such as degree, were computed as the total number of interactions in which a protein is involved, while betweenness and closeness centralities were computed using the NetworkX Python library^18^.

### General gene properties

We used general gene properties in addition to the properties compiled from the sources above. Properties directly extracted from Ensembl Biomart Genes^16^ were: Gene % GC content, Transcript count and Gene Length, while some were calculated: StdDev Transcript length, Average Transcript length, Min Transcript length, Max Transcript length and Exon Count. A list of all human Ohnolog genes with strict and intermediate score was downloaded from this database: (http://ohnologs.curie.fr/).

### Machine learning method

To build a supervised machine-learning model, we need to identify what the model is trying to predict and add that outcome to every row in the dataset. The outcome in our dataset is binary (true or false), indicating whether a gene has been identified as a cancer gene or not. This was done by seeing whether the gene had been added to the COSMIC Cancer Gene Census. A heuristic logic was used to find the best performing model. The best modelling algorithm and configuration were selected based on a performance metric. For our model, this was logistic loss. The top performing model was Gradient Boosting. This model achieved the best logistic loss across both training and validation datasets.

To avoid over-fitting, the best practice is to evaluate model performance on out-of-sample data. If the model performs very well on in-sample data, (the training data), but poorly on out-of-sample data, that is an indication that the model is over-fit. The k-fold cross-validation is a standard technique used to validate model performance and ensure that over-fitting does not occur. We used a fivefold cross-validation framework as the default option to test the out-of-sample stability of a model's performance. In addition to the cross-validation partitioning, a holdout sample (test sample) is used to further test out-of-sample model performance, thus ensuring appropriate evaluation of the model performance and reducing likelihood of over-fitting. 20% of the training data was set aside as a holdout dataset. This dataset is used to verify that the final model performs well on data that has not been touched throughout the training process, while the remainder of the data is divided into 5 cross validation partitions. Because the distribution of the target’s values in a binary classification may be imbalanced, the validations' partitions were randomly selected using a stratified sample approach where sub-populations within the data are always represented in each partition to preserve the distribution of the target’s values for each partition. Our selected model algorithm is Gradient Boosting Machines (or Generalized Boosted Models, ‘GBM’). GBM is a cutting-edge algorithm for fitting extremely accurate predictive models^19^. GBMs are a generalisation of Freund and Schapire’s adaboost algorithm (1995) modified to handle arbitrary loss functions. They are similar in concept to random forests, in that they fit individual decision trees to random re-samples of the input data, where each tree sees a bootstrap sample of the rows of the dataset and N arbitrarily chosen columns, where N is a configurable parameter of the model. GBMs differ from random forests in a single major aspect: rather than fitting the trees in parallel, the GBM fits each successive tree to the residual errors from all the previous trees combined. This is advantageous, as the model focuses each iteration on the examples that are most difficult to predict (and therefore most useful to get correct). Due to their iterative nature, GBMs are almost guaranteed to over-fit the training data, given enough iterations. The two critical parameters of the algorithm, therefore, are the learning rate (or how fast the model fits the data) and the number of trees the model is allowed to fit. It is critical to cross-validate these two parameters. When done correctly, GBMs are capable of finding the exact point in the training data where over-fitting begins, and halts one iteration prior to that. In this manner, GBMs are usually capable of producing the model with the highest possible accuracy without over-fitting. Our model uses logistic loss and early stopping to determine the best number of trees. Early stopping is a method for determining the number of trees to use for a boosted trees model. The training data is split into a training set and a validation set; in each iteration the model is scored using the validation set. If validation set performance decreases for 200 iterations, the training procedure stops, and the model returns the fit at the best tree seen so far. Note that the early stopping validation set will be a 90/10-train/validation split within the training data for a given model. The model will therefore internally use 90% of the available training dataset and 10% of the data for early stopping. Since the early stopping test set was used to find the optimal termination point, it cannot be used for training. Several guardrails were implemented to mitigate possibilities of data labelling bias. We ensured the dataset used for training does not carry any overrepresentation for any feature or group. Another common machine learning pitfall is the possibility of ‘leakage’ where a feature used in the training data would not be fully known until the outcome has occurred. This could create a false level of accuracy. Such correlations should be detected and eliminated before the model is built. A leakage detection method based on the GiniNorm metric was implemented, thus ensuring that there was no leakage in our model.

To address the imbalanced dataset issue, a technique called ‘Smart Downsampling’ was used during the model preparation stage. The majority class of the dataset (in this case the genes that are not confirmed to be cancer-associated) is down-sampled during the preparation stage to reach a balanced dataset, then a weight is added so that the effect of the resulting dataset mimics the original balance of the classes without losing any embedded signal. The smart downsampling technique ensure that the accuracy of the model is not affected by variation in the number of samples in different classes and accounts for class imbalance by stratifying the sample by class. In our model, the entire minority class is preserved (a weight of 1 is applied) and sampling was only applied to the majority class. Because accuracy is typically more important on the minority class, this technique preserves model accuracy well. Furthermore, the model’s optimisation metric used to identify the most accurate model is LogLoss. As LogLoss is an error metric which penalizes wrong predictions, it is therefore suitable to use with downsampled or imbalanced datasets.

Numeric missing values are imputed with an arbitrary value (− 9999). For categorical variables, missing values are treated as an additional level in the categories. To set the hyperparameters used by the model, a grid search was performed to ensure optimum accuracy using the selected hyperparameters values within a reasonable execution timeframe. Accuracy related information for our model, such as the ROC curve, sensitivity, and specificity, are discussed in the “results” section.

## Results

### Cancer-associated genes and essentiality scores

We first determined whether cancer-related genes are likely to have high essentiality scores. We aggregated several essentiality scores calculated by multiple metrics^5^ for the list of genes identified in the COSMIC Census database (Oct 2018) and for all other human protein coding genes. Two different approaches to scoring genes’ essentiality are available. The first group of methods calculates the essentiality scores by measuring the degree of loss of function caused by a change (represented by variation detection) in the gene. It uses the following methods: residual variation intolerance score (RVIS), LoFtool, Missense-Z, the probability of loss-of-function intolerance (pLI) and the probability of haplo-insufficiency (Phi). The second group (Wang, Blomen and Hart- EvoTol) studies the impact of variation on cell viability. For all methods above measuring essentiality, a higher score indicates a higher degree of essentiality. Each method is described in detail in^5^.

We find that on average the cancer genes exhibit a higher degree of essentiality compared to the average scores calculated for all protein coding human genes and all metrics (Fig. 1). We find that genes associated with cancer have higher essentiality scores on average in both categories (intolerance to variants and cell line viability), compared to the average scores across all human genes. *P* values are consistently < 0.00001 (Table 1).

We also investigated whether Tumor Suppressor Genes (TSGs) or Oncogenes as distinct groups of genes would show different degrees of essentiality. (If the gene is known to be both an oncogene and a TSG, then the essentiality score of that gene would be present in both the oncogene and the TSG groups). We found no significant differences in the degrees of essentiality on average for either group compared to the set of all cancer genes (Table 1; Fig. 1).

The results are particularly of interest in the context of cancer, as essential genes have been shown to evolve more slowly than nonessential genes^20–22^, although some discrepancies have been reported^22^. A slower evolutionary rate indicates less probability to evolve resistance to a cancer drug. This is particularly important in the case of anticancer drugs as it was reported that these drugs cause a change in the selection pressure when administered, leading to increased drug resistance^23^.

### Cancer-associated genes prediction analysis results

This association between cancer-related genes and essentiality scores prompted us to develop methods to identify cancer-related genes using this information. We used a machine-learning approach. A range of open-source algorithms were applied and tested to produce the most accurate classifier. We focus on properties related to protein–protein interaction networks, as essential genes are likely to encode hub proteins, i.e., those with highest degree values in the network^21,24^.

A total of nine different modelling approaches (or configurations) were run on the data to ensure the selection of the best performing approach (the list of these can be found in the Supplementary Information Table 2, along with their performance metrics). The performance metric used to rank the models was Logarithmic Loss (LogLoss), LogLoss is an appropriate and known performance measure when the model is of a binary-classification type. The LogLoss measures confidence of the prediction and estimates how to penalise incorrect classification. The selection mechanism for the performance metric takes the type of model (binary classification in this case) and distribution of values into consideration when recommending the performance metric. However, other performance metrics were also calculated (Supplementary Information Table 2). The performance metrics are calculated for all validation and test (holdout) sets to ensure that the model is not over-fitting. The particular model with best performance result (LogLoss) in this case was: eXtreme Gradient Boosted Trees Classifier with Early Stopping. The model shows very close LogLoss values for training/validation and holdout data sets (Table 2), demonstrating no over-fitting.

**Table 2:** The LogLoss scores for our model validations and holdout segments.

Scoring type	Score (LogLoss)
One validation	0.097
Cross validation (average of all sets)	0.098
Holdout	0.099

**Figure 2:**
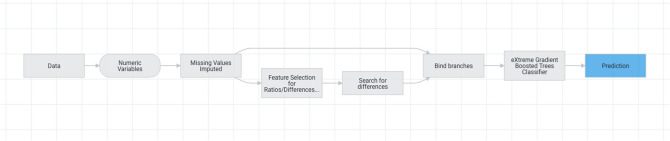
Model development stages.

The model development workflow (i.e., the model blueprint) is shown in Fig. 2. This shows the pre-processing steps and the algorithm used in our final model, and illustrates the steps involved in transforming input into a model. In this diagram, ‘Ordinal encoding of categorical variables’ converts categorical variables to an ordinal scale while the ‘Missing Values Imputed’ node imputes missing values. Numeric variables with missed values were imputed with an arbitrary value (default − 9999). This is effective for tree-based models, as they can learn a split between the arbitrary value (− 9999) and the rest of the data (which is far away from this value).

**Figure 3:**
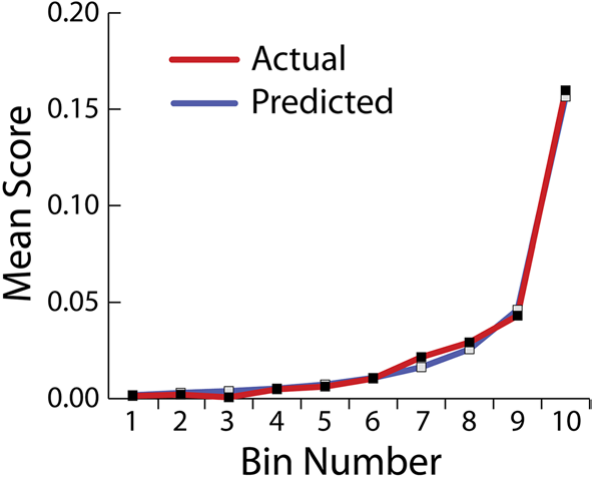
The Lift Chart illustrating the accuracy of the model.

To demonstrate the effectiveness of our model, a chart was constructed (Fig. 3) that shows across the entire validation dataset (divided into 10 segments or bins and ordered by the average outcome prediction value) the average actual outcome (whether a gene has been identified as cancer gene or not) and the average predicted outcome for each segment of the data (order from lowest average to highest per segment). The left side of the curve indicates where the model predicted a low score on one section of the population while the right side of the curve indicates where the model predicted a high score. The "Predicted" blue line displays the average prediction score for the rows in that bin. The "Actual" red line displays the actual percentage for the rows in that bin. By showing the actual outcomes alongside the predictive values for the dataset, we can see how close these predictions are to the actual known outcome for each segment of the dataset. Also, we can determine if the accuracy diverges in cases where the outcome is confirmed as cancer or not, as the segments are ordered by their average of outcome scores.

In general, the steeper the “actual” line is, and the more closely the “predicted” line matches the actual line, the better the model. A close relationship between these two lines is indicative of the predictive accuracy of the model; a consistently increasing line is another good indicator of satisfactory model performance. The graph we have for our model (Fig. 3) thus indicates high accuracy of our prediction model.

In addition, the confusion matrix (Table 3) and the summary statistics (Table 4) show the actual versus predicted values for both true/false categories for our training dataset (80% of the total dataset). The model statistics show the model reached just over 89% specificity and 60% sensitivity in predicting cancer genes. This means that we are able to detect over half of cancer genes successfully while only misclassifying around 10% of non-cancer genes within the training/validation datasets. The summary statistics (Table 4) also shows the F1 score (harmonic mean of the precision and recall) and Matthews Correlation Coefficient (MCC is the geometric mean of the regression coefficient) for the model. The low F1 score reflects our choice to maximise the true negative rate (preventing significant misclassification of non-cancer genes).

**Table 3:** The model’s confusion matrix (where TP is true positives. TN is true negatives. FP is false positives. FN is false negatives).

		Predicted		
Actual		−	+	
	−	12,493 (TN)	1490 (FP)	**13,983**
	+	159 (FN)	243 (TP)	**402**
		**12,652**	**1733**	

Bold figures are sums of the rows and columns.

**Table 4:** Summary of the model’s performance statistics.

F1 score	True positive rate (Sensitivity)	False positive rate (Fallout)	True negative rate (Specificity)	Positive predictive value (Precision)	Negative predictive value	Accuracy	Matthews correlation coefficient
0.23	0.61	0.11	0.89	0.14	0.99	0.89	0.25

### The false positives

To further confirm the model’s ability to predict cancer genes, we used the model on 190 new cancer genes that had been added to the COSMIC’ Cancer Census Genes between October 2018 and April 2020. Applying the model, we were able to predict 56 genes out of the newly added 190 genes as cancer genes, all of which were among the false positives detected by the model. This indicates that the model is indeed suitable to use to predict novel candidate cancer genes that could be experimentally confirmed later. A full ranked list of candidate genes predicted to be cancer associated by our model is available in Supplementary Information Table 3.

**Figure 4:**
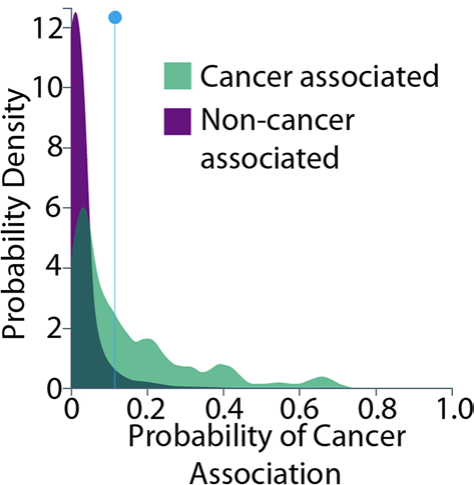
The prediction distribution graph showing how well the model discriminates between cancer and non-cancer genes.

Another way to visualise the model performance, and determine the optimal score to use as a threshold between cancer and non-cancer genes, is the ‘prediction distribution’ graph (Fig. 4) which illustrates the distribution of outcomes. The distribution in purple shows the outcome where gene is not classified as a cancer gene while the second distribution in green shows the outcomes where gene is classified as a cancer gene. The dividing line represents the selected threshold at which the binary decision creates a desirable balance between true negatives and true positives. Figure 4 shows how well our model discriminates between prediction classes (cancer gene or non-cancer gene) and shows the selected score (threshold) that could be used to make a binary (true/false) prediction for a gene to be classified as a candidate cancer gene. Every prediction to the left of the dividing line is classified as non-cancer associated and every prediction to the right of the dividing line is classified as cancer associated.

The prediction distribution graph can be interpreted as follows: purple to the left of the threshold line is for instances where genes were correctly classified as non-cancer (true negatives). Green to the left of the threshold line is for instances were incorrectly classified as non-cancer (false negatives). Purple to the right of the threshold line, is for instances that were incorrectly classified as cancer gene (false positives). Green to the right of the threshold line, is for instances were correctly classified as cancer genes (true positives). The graph again confirms that the model was able to accurately distinguish cancer and non-cancer genes.

**Figure 5:**
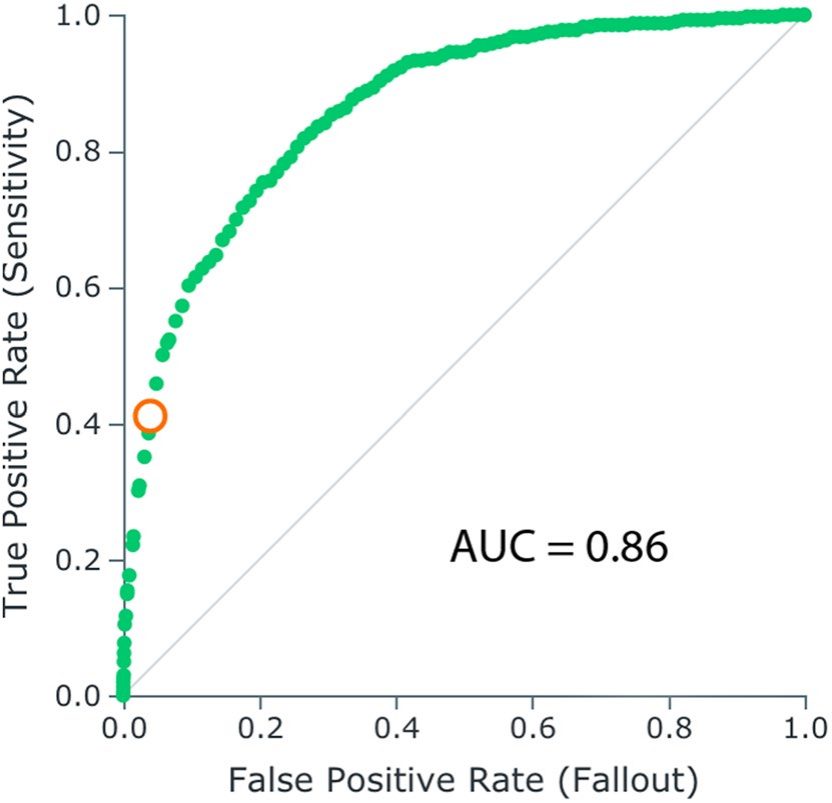
The receiver operator characteristic (ROC) curve indicating model performance.

Using the receiver operating characteristic curve (ROC) curve produced for our model (Fig. 5), we were able to evaluate the accuracy of prediction. The AUC (area under the curve) is a metric for binary classification that considers all possible thresholds and summarizes performance in a single value, with the larger the area under the curve, the more accurate the model. An AUC of 0.5 shows that predictions based on this model are no better than a random guess. An AUC of 1.0 shows that predictions based on this model are perfect. (This is highly uncommon and likely flawed, indicating some features that should not be known in advance are being used in model training and thus revealing the outcome.) As the area under the curve is of 0.86, we conclude that the model is accurate. The circle intersecting the ROC curve represents the threshold chosen for classification of genes. This is used to transform probability scores assigned to each gene into binary classification decisions, where each gene would be classified as a potential cancer gene or not.

### Feature impact

Feature impact measures how much worse a model’s error score would be if the model made predictions after randomly shuffling the values of one field input (while leaving other values unchanged) and thus shows how useful each feature is for the prediction. The scores were normalised so that the value of the most important feature column is 100% and the other subsequent features are normalised to it. This helps identify those properties that are particularly important in relation to predicting cancer genes and would aid in further our understanding of the biological aspects that might underline the propensity of a gene to be a cancer gene.

**Figure 6:**
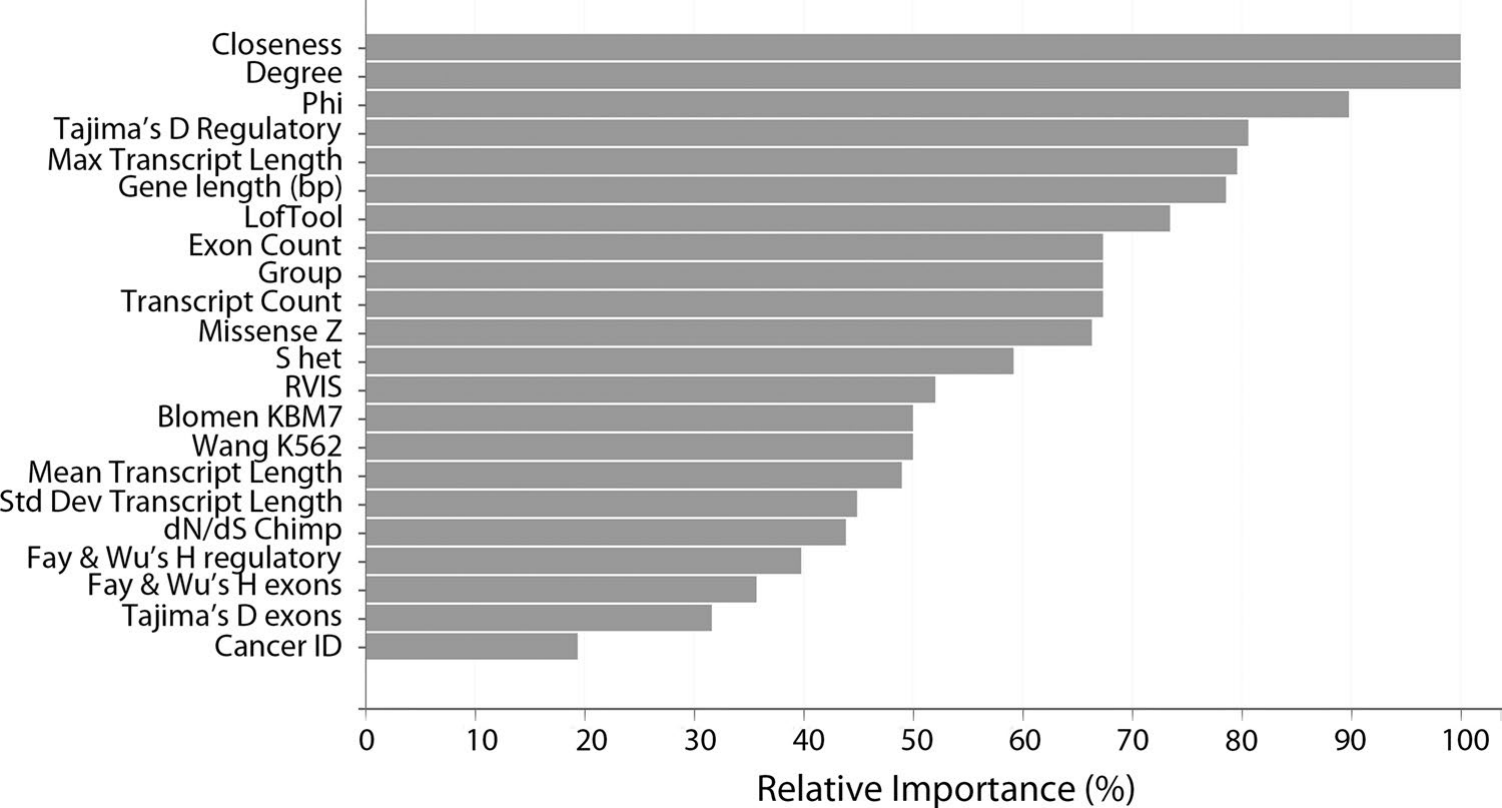
The top properties ranked by their relative importance used to make the predictions by the model.

‘Closeness’ and ‘degree’ are ranked as the properties with the highest feature impact (Fig. 6). Both are protein–protein interaction network properties, indicating a central role of the protein product within the network. We find that both correlate with likelihood of cancer association. Other important properties such the ‘phi’ essentiality score (probability of haploinsufficiency compared to baseline neutral expectation) and Tajima’s D regulatory (measures for genetic variation at intra-species level and for proportion of rare variants) show that increased essentiality accompanied with occurrence of rare variants increase the likelihood of pathological impact and for the gene to be linked to cancer initiation or progression. We also note that greater length of a gene or transcript increases the likelihood of a somatic mutation, so increasing the chance of a mutation within that gene, thus increasing the likelihood of it being a cancer gene.

To confirm that the selected model performance is optimal based on the input data used, we created a new blended model combining the best 2nd and 3rd modelling approaches from all modelling approaches tested within our project and compared the performance metric (AUC) of our selected model with the new blended model. We found that improvement is small (0.008), despite the added complexity, where the blended model achieved an AUC of 0.866 and our single selected model achieved an AUC of 0.858.

We have also retrained our model using a dataset that excludes general gene properties and found that a reduction in model’s performance was evident but very small. The model trained on this dataset achieved an AUC of 0.835 and a sensitivity of 55% at a specificity of 89%. This small reduction in the predictability of the models indicates that essentiality and protein–protein interaction network properties are the most important features predicting cancer genes and that information carried by gene general properties can be in most part be represented by information carried by these properties. This can be rationalised, as longer genes (median transcript length = 3737) tend to have the highest number of protein–protein interactions^25^.

### Comparison with other cancer driver genes prediction methods

According to a recent comprehensive review of cancer driver genes prediction models, currently the best performing machine learning model is driverMAPS with an AUC of 0.94, followed by HotNet2 with an AUC of 0.81^4^. When comparing our model performance using AUC to the other 12 reviewed cancer driver genes prediction models, our model would come second with an AUC of 0.86. Our predictive model achieved better AUC measured performance when compared to the best model that used a similar network based approach (HotNet2 with AUC = 0.81) and better than the best function-based prediction model (MutPanning with AUC = 0.62). The strong performance of our model indicates the importance of combining different and distinctive gene properties, when building prediction models, while avoiding reliance on the frequency approach that could mask important driver genes that were detected in fewer samples. Despite the apparent success and high AUC score reported by our model, this should be treated with some caution. The AUC value is based on the ROC curve which is constructed by varying the threshold and then plotting the resulting sensitivities against the corresponding false positive rates. Several statistical methods are available to use to compare two AUC results and determine if the difference is significant^26–28^. These methods require the ranking of the variables in its calculations (e.g., to calculate the variance or covariance of the AUC). The ranking of predicated cancer associated genes was not available from all the other 12 cancer driver genes prediction methods. Thus, we were not able to determine whether the difference between the AUC score of our method and the AUC scores of these methods is significant.

The driverMAPS (Model-based Analysis of Positive Selection) method (the only method with higher AUC compared to our model) identifies cancer candidate genes using the assumption that these genes would exhibit elevated mutation rates in functionally important sites^29^. Thus, driverMAPS combines frequency- and function-based principles. Unlike our model that uses certain cohorts of genes properties, the parameters used in driverMAPS are mainly derived and estimated from factors influencing positive selection on somatic mutations. However, there are few features in common between the two models, such as dN/dS.

Despite driverMAPS had the overall best performance, network-based methods (like our method) showed much higher sensitivity than driverMAPS therefore potentially making more them more suited to distinguish cancer driver from non-driver genes. The driverMAPS paper^29^ provides a list of novel driver genes. We found that 35% of these novel candidate genes were also predicted by our model. Differenced in genes identified as cancer-related in the two approaches could be attributed to the different nature of features used by the two models. We believe that there is evidence^30^ pointing to genes with low mutation rates, but with important roles in driving the initiation and progression of tumours. Genes with high mutation rates were also shown to be less vital than expected in driving tumor initiation^31^. This variability in the mutation rate correlation with identified driver genes might explain some genes that our model does not identify as cancer-related genes where driverMaps does. Our model uses properties that are available for most protein coding genes, while driverMaps applies to genes already identified in tumour samples and predicts their likelihood to be driver cancer genes. Thus, the candidate list of genes provided by driverMaps is substantially smaller than our list. Using an ensemble method that evaluates both driverMAPS score and our models’ score for each gene, may produce more a reliable outcome. This would require further validation.

### The cancer genes association with whole genome duplications and Ohnologs

Enriching the model’s training dataset with added properties that show correlations with oncogenes could enhance the model prediction ability and elevate further the accuracy of the model. One potential feature is knowing whether a gene is an Ohnolog gene.

Paralogs retained from whole genome duplications (WGD) events that have occurred in all vertebrates, some 500 Myr ago are called ohnologs after Susumu Ohno^32^. Ohnologs have been shown to be prone to dominant deleterious mutations and frequently implicated in cancer and genetic diseases^32^. We investigated the enrichment of ohnologs within cancer-associated genes. Ohnolog genes can be divided into three sets: strict, intermediate and relaxed. These three sets are constructed using statistical confidence criteria^32^ . We found that 44% of the total number of cancer-associated genes (as reported in COSMIC census) belongs to an ohnolog family (using strict and intermediate thresholds). Considering that 20% of all known human genes are ohnologs (strict and intermediate) and that cancer-associated genes comprise less than 4% of all human genes, the enrichment of ohnolog genes with cancer-related genes is two times higher than expected. If only ohnologs that pass the strict threshold were considered, the fraction of cancer-related genes that are ohnologs is still high at 34%.

When performing pathway analysis (carried out using PANTHER gene ontology release 17.0), we found that cancer associated ohnologs show statistically significant enrichment (> tenfold) in many pathways and particularly within signalling pathways known to be cancer associated such as Jak/STAT, RAS and P53 (Supplementary Information Table 4). On the other hand, ohnologs that are not cancer associated are present in fewer signalling pathways and at enrichment (< fourfold) and in various other pathways that we do not see for cancer associated ohnologs (Supplementary Information Table 5). There is evidence the major components and capabilities of cell signalling pathways arose with, or shortly after, the of origin of metazoans, and that ability to form growth factor gradients evolved after ctenophores split from the rest of the metazoa^33^. Molecular clocks and objective fossil records suggest that the early metazoans were in existence at ∼550–600 Myr ago^34,35^ which coincides roughly with the whole genome duplication events that produced the ohnologs found to be cancer associated.

## Discussion

Cancer is a complex disease; research that worked on providing the genomic profile of the disease is still producing new findings. For example, the number of genes implicated in cancer increased by over 30% in the last 4 years as per the COSMIC genes census^36^. Identifying how cancer-associated genes differ from non-cancer related genes would allow us a better understanding of the disease and could enhance the usage of advanced prediction analysis in finding genes that are implicated in tumor initiation and progression (drivers).

Using machine learning techniques has great potential in producing accurate predictive models that could indicate the likelihood of a gene or a mutation to be cancer-associated, highlighting at the same time the relationship between the properties used in the model and the prediction. Machine learning techniques are often superior to traditional statistical methods because they are more flexible and rely on fewer statistical assumptions. The only assumption being made is that the model training data is representative of the future scoring data.

Here we contributed to the ongoing efforts in predicting cancer associated genes. We showed that combining various properties of cancer genes, including evolutionary related measures such as selection pressure and measures of genetic variants, to train a machine-learning model to identify cancer related genes could result in superior model performance. One property that was not investigated before in relation to predicting cancer associated genes is the essentiality of the gene. As mutated cancer-associated genes generally do not compromise viability in a direct manner, it might be expected that it is unlikely for these genes to score high on the essentiality spectrum. However, we demonstrated that on average there is a positive correlation between gene essentiality and cancer. We applied a range of methods that score the degree of essentiality. In particular, we applied LofTool and Missense Z-score where the calculation of essentiality scores is based on intolerance to variants in human population sequenced data, and Blomen KBM7 and Wang K562, where cell viability data is used. We found that the cancer-associated genes exhibit a higher degree of essentiality compared to the scores calculated for all protein coding human genes. Our results could be interpreted in the context of the genes’ involvement in particular biological activities. Genes classed as essential are often involved in cell, embryo, and organism growth. Similarly, proliferation is key for cancer cells. Therefore, the sets of genes that are essential and those that are involved in unregulated growth, as seen in cancer, tend to overlap. This finding provides further evidence for the importance of evolutionary aspects when studying cancer genes. Scientists might be able to further the understanding of cancer by incorporating properties linked to essentiality in their studies. Several previous studies looked at the relationship between evolutionary conservation and the degree of essentiality in genes across species^7,20^. Essential genes have been shown to be more conserved and to evolve more slowly than nonessential genes in human^7,20^. We hypothesise that cancer-associated genes that are highly essential could be more suitable candidates for targeted therapies, potentially providing less likelihood of developing drug resistance due their increased conserved status. It has been shown that cancer drugs cause a change in the selection pressure when administered, leading to increased drug resistance^23^, so if the essential nature of a gene could slow its ability to evolve drug resistance, then these genes should prioritised for drug discoveries when possible.

This result prompted us to develop a machine-learning model that could predict cancer-associated genes using essentiality related and general genomic properties; we extended the range of gene properties in our dataset to include, in addition to the essentiality scores, properties strongly linked to (although do not directly a measure of) the gene’s essentiality. Essential genes are likely to encode hub proteins in protein–protein interaction networks, have smaller-sized introns, are abundant and are ubiquitously expressed in cells and tissues^6^. It was shown too that the more essential the gene is, the smaller the number of reported missense mutations for this gene^5^. Therefore, in addition to general gene properties, like gene % GC content and transcript count, we added protein–protein interaction network properties, such as degree indicating the number of interactions, closeness and betweenness. We also added various measures indicating selection pressure, such as dN/dS and measures of genetic variants, such as Tajima's D, based on exons and regulatory sequences, and Fay and Wu's H, based on exons and regulatory sequences.

We tested different model configurations, selecting the model with the best performance. The resulting classifier displays excellent performance in predicting whether a human protein-coding gene is cancer-related; it achieved 89% for the accuracy and the AUC was > 0.85. Our machine-learning model prediction scores provide a good base to prioritise the likelihood of a human protein coding gene to be a cancer gene. Of key importance in our results are those predictions that are false positives, i.e., those genes with high scores that have no published cancer association. Two possible explanations exist: either they represent a failure of the model to correctly classify the data or, alternatively, these gene are in fact cancer related, but have not yet been characterised as such. These genes are therefore likely to encode future cancer targets.

The set of features that can be used to train a machine learning model to predict cancer-associated genes could be expanded further to include other features. For example, a feature that indicates whether a gene is an Ohnolog could potentially elevate the model accuracy even further.

Our machine-learning model identified the most important properties for the classification, ranking the properties by their impact on the prediction and revealing their influences on the genes found to be cancer associated. Protein–protein interactions properties, such as degree and closeness, are confirmed to be very influential when assessing the likelihood of a gene to be cancer-associated. This reflects that cancer-associated genes often code for protein found in hubs within the protein–protein interaction networks. The ranking also showed essential score Phi and Tajima’s D Regulatory to be among top impactful features (albeit to lesser extent). This confirms that on average these genes are more essential than other non-cancer genes and shows evidence for positive selection on these genes. These findings may offer targets for further research.

According to a recent comprehensive review of cancer driver genes prediction models^4^, currently the best performing machine learning model is driverMAPS with AUC = 0.94 followed by HotNet2 with AUC = 0.81. When comparing our model performance to the other 12 reviewed cancer driver genes prediction models using purely the AUC, our model would come second with AUC = 0.86. Our predictive model achieved a better AUC measured performance when compared to the top-performing model using a similar network-based approach (HotNet2 with AUC = 0.81) and better than the best function-based prediction model (MutPanning with AUC = 0.62). This potential strong performance (a statistical test is required to confirm whether these differences in AUC scores are significant) may indicate the importance of combining different and distinctive gene properties when building prediction models. Combining the most accurate classifiers could lead to a powerful predictor that can ensure that fewer genes are misclassified. Our work provides a good basis for scientists to start considering novel candidate genes predicted to be cancer-associated in their research. Cancer genes databases such as COSMIC could also incorporate these candidate cancer-associated genes (possibly in a separate tier) and make them available for researchers.

### Supplementary Information

Supplementary Information 1.

Supplementary Information 2.

Supplementary Information 3.

Supplementary Information 4.

Supplementary Information 5.

Supplementary Information 6.

## Data Availability

All data generated or analysed during this study are included in this published article and its Supplementary Information files.

## Code availability

The code generated during this study is included in the Supplementary Information.
