# Supplementary material for: Essentiality, protein–protein interactions and evolutionary properties are key predictors for identifying cancer-associated genes using machine learning
Source: Sci Rep. 2024 Apr 22;14:9199. doi: 10.1038/s41598-023-44118-2 (PMC11035574; doi:10.1038/s41598-023-44118-2)
Supplement: Supplementary file 1 — Supplementary Information 1. [file 41598_2023_44118_MOESM1_ESM.docx]

**Cancer Genes Prediction SI Code**

Amro Safadi, Simon C. Lovell & Andrew J. Doig

The code (in Python programming language) could be run as a stand alone code to execute predictions based on the machine-learning model we built to predict Cancer-associated genes using on protein-protein interaction networks, essentiality scores and evolutionary properties:

import calendar

from datetime import datetime

from collections import namedtuple

import re

import sys

import time

import os

import numpy as np

import pandas as pd

PY3 = sys.version_info[0] == 3

if PY3:

string_types = str,

text_type = str

long_type = int

else:

string_types = basestring,

text_type = unicode

long_type = long

def predict(row):

Group = row[u'Group']

round_Average_Transcript_length = np.float32(row[u'Average Transcript length'])

round_Blomen_KBM7 = np.float32(row[u'Blomen KBM7'])

round_Blomen_KBM7_mi = np.float32(row[u'Blomen KBM7-mi'])

round_Closeness = np.float32(row[u'Closeness'])

round_Degree = np.float32(row[u'Degree'])

round_Degree_mi = np.float32(row[u'Degree-mi'])

round_End = np.float32(row[u'End'])

round_Exon_Count = np.float32(row[u'Exon Count'])

round_Gene_Length_bp = np.float32(row[u'Gene Length bp'])

round_LofTool = np.float32(row[u'LofTool'])

round_LofTool_mi = np.float32(row[u'LofTool-mi'])

round_Phi = np.float32(row[u'Phi'])

round_Phi_mi = np.float32(row[u'Phi-mi'])

round_StdDev_Transcript_length = np.float32(row[u'StdDev Transcript length'])

round_Tajima__s_D_regulatory = np.float32(row[u'Tajima\'s D regulatory'])

round_Tajima__s_D_regulatory_mi = np.float32(row[u'Tajima\'s D regulatory-mi'])

round_Transcript_count = np.float32(row[u'Transcript count'])

round_dN_dS_Chimp = np.float32(row[u'dN/dS Chimp'])

round_dN_dS_Chimp_mi = np.float32(row[u'dN/dS Chimp-mi'])

round_missense_Z = np.float32(row[u'missense_Z'])

round_missense_Z_mi = np.float32(row[u'missense_Z-mi'])

round_s_het = np.float32(row[u's_het'])

round_s_het_mi = np.float32(row[u's_het-mi'])

return sum([

-2.6863578,

0.018202720175283508552 * (not Group == u'CM' and

not Group == u'MNC' and

round_Tajima__s_D_regulatory <= 0.4104999899864197 and

round_StdDev_Transcript_length > 1954.456787109375),

-0.0054378635982185617379 * (round_dN_dS_Chimp_mi <= 0.5 and

round_Blomen_KBM7 <= -0.2516007423400879 and

round_LofTool <= 0.6634999513626099 and

round_LofTool_mi <= 0.5),

-1.5583491431925192728E-11 * (round_End),

0.048499210437713213828 * (round_Closeness <= 0.33500000834465027 and

3.2976694107055664 < round_missense_Z <= 4.050085067749023),

0.096975931123053304983 * (round_Transcript_count > 20.5),

-0.017383948168761587799 * (round_Degree <= 3.5 and

round_s_het > 0.023678744211792946),

-0.0081188248086857105895 * (not Group == u'CM' and

round_Tajima__s_D_regulatory <= 0.17550000548362732 and

round_Transcript_count <= 10.5 and

round_Gene_Length_bp > 2814.5),

-0.023776888724096147121 * (round_Tajima__s_D_regulatory > -1.2874999046325684 and

round_missense_Z <= 3.2976694107055664 and

round_missense_Z_mi <= 0.5 and

round_LofTool <= 0.6634999513626099),

0.015303739394473513113 * (round_LofTool > 0.6634999513626099 and

round_Gene_Length_bp > 104704.0 and

round_StdDev_Transcript_length > 2574.27734375 and

round_Exon_Count <= 156.5),

0.032356712896822563408 * (round_Degree_mi <= 0.5 and

0.874500036239624 < round_LofTool <= 0.9921150207519531),

-0.061064130313854887711 * (not Group == u'MNC' and

round_dN_dS_Chimp_mi > 0.5 and

round_missense_Z <= 3.2976694107055664 and

round_StdDev_Transcript_length <= 987.5745849609375),

0.03107445629333745532 * (round_Phi > 0.00015248148702085018 and

round_Blomen_KBM7 > -0.5508977174758911 and

round_missense_Z <= 4.0604472160339355 and

round_Exon_Count > 87.5),

0.027000278790342342738 * (round_Degree <= 12.5 and

round_s_het > 0.017613736912608147 and

round_Gene_Length_bp > 38001.5 and

round_Exon_Count <= 221.5),

-0.0074861761526758031915 * (round_Tajima__s_D_regulatory <= 0.4165000021457672 and

round_LofTool > 0.6634999513626099 and

round_Transcript_count <= 21.5 and

round_StdDev_Transcript_length <= 2558.05078125),

0.033691518811985399218 * (round_Blomen_KBM7 <= -0.14686328172683716 and

round_LofTool > 0.962399959564209 and

round_StdDev_Transcript_length <= 2391.52490234375),

-0.016812786172447410221 * (round_Degree_mi > 0.5 and

round_Phi_mi <= 0.5 and

round_Blomen_KBM7 > -0.27386000752449036 and

round_Blomen_KBM7_mi <= 0.5),

-0.05868424164045044078 * (round_Degree > 29.5 and

round_StdDev_Transcript_length <= 897.559326171875),

0.014660818041939896808 * (round_Degree > 17.5 and

round_Degree_mi <= 0.5 and

round_LofTool > 0.9311000108718872),

0.0031378779289614714028 * (round_Degree > 5.5 and

round_Tajima__s_D_regulatory <= 0.44699999690055847 and

round_Blomen_KBM7 <= -0.2522552013397217 and

round_Average_Transcript_length > 2566.631103515625),

0.049576634648417938767 * (round_Closeness > 0.3149999976158142 and

round_Blomen_KBM7 > -0.5158457159996033 and

round_Gene_Length_bp <= 53140.5),

0.033125285580605914881 * (round_Degree > 56.5 and

round_Closeness > 0.2549999952316284 and

round_Gene_Length_bp > 41216.0),

0.015426516511084873567 * (not Group == u'CM' and

round_Tajima__s_D_regulatory > 0.4235000014305115 and

round_s_het <= 0.014262920245528221),

-0.054664675004081307585 * (round_Closeness > 0.3149999976158142 and

round_s_het <= 0.025150161236524582),

0.064971575636202566484 * (round_Degree > 12.5 and

round_Blomen_KBM7 <= -0.17924460768699646 and

round_Gene_Length_bp > 38001.5),

0.008076281326937726282 * (round_End <= 100419720.0 and

round_Tajima__s_D_regulatory > -1.2885000705718994 and

round_Blomen_KBM7 <= -0.18547898530960083 and

round_missense_Z <= 3.2976694107055664),

-0.0025333307179157250021 * (not Group == u'CM' and

round_Tajima__s_D_regulatory <= -1.2894999980926514 and

round_Blomen_KBM7 <= -0.5147985219955444 and

round_Transcript_count <= 26.5),

0.020805097212741045093 * (not Group == u'NDNE' and

round_Degree_mi <= 0.5 and

0.659500002861023 < round_LofTool <= 0.9907699823379517),

0.0013239217591041027491 * (round_Closeness > 0.3149999976158142 and

round_Phi <= 0.0015477617271244526),

0.19073466683054091098 * (round_Closeness > 0.3149999976158142 and

round_Blomen_KBM7 > -0.5528146028518677 and

round_Exon_Count <= 224.5),

0.010314188977423621382 * (round_End > 127204736.0 and

round_Tajima__s_D_regulatory <= 0.4599999785423279 and

round_Transcript_count > 10.5 and

round_Gene_Length_bp > 2814.5),

-0.069447321340016812674 * (round_Degree_mi > 0.5),

0.016914894808412236221 * (Group == u'MNC' and

round_dN_dS_Chimp_mi <= 0.5 and

round_Blomen_KBM7 > -0.5146373510360718),

0.027726420391460625953 * (round_Degree <= 3.5 and

round_StdDev_Transcript_length <= 1049.45654296875),

-0.0021865984701677676667 * (not Group == u'MNC' and

round_Degree <= 62.5 and

round_Degree_mi <= 0.5 and

round_Blomen_KBM7 <= -0.2516775131225586),

0.0053561320466416163441 * (Group == u'MNC' and

round_dN_dS_Chimp_mi <= 0.5 and

round_s_het > 0.028055116534233093 and

round_StdDev_Transcript_length > 168.6014404296875),

0.11425430800029053036 * (not Group == u'NDNE' and

round_Degree > 3.5 and

round_Closeness > 0.3149999976158142 and

round_Gene_Length_bp > 41501.0),

-0.0032500288883767361817 * (round_Tajima__s_D_regulatory <= 1.4165000915527344 and

round_Blomen_KBM7 > -0.5153244733810425 and

round_StdDev_Transcript_length <= 166.56736755371094),

0.028508029551613675578 * (not Group == u'MNC' and

round_missense_Z <= 2.642360210418701 and

round_s_het <= 0.014316117390990257),

-0.0088550958230132672394 * (round_Closeness <= 0.33500000834465027 and

round_dN_dS_Chimp_mi > 0.5 and

round_Tajima__s_D_regulatory <= 0.4104999899864197 and

round_Exon_Count <= 97.5),

0.051816337733702685919 * (not Group == u'NDNE' and

round_Degree > 12.5 and

round_StdDev_Transcript_length > 636.7213134765625 and

round_Exon_Count <= 253.5),

-0.00060243449056097606292 * (round_Tajima__s_D_regulatory),

0.068946378006664615912 * (round_Transcript_count > 26.5),

-0.059757348541248728191 * (round_Degree_mi <= 0.5 and

round_Closeness > 0.3050000071525574 and

round_missense_Z_mi <= 0.5 and

round_Gene_Length_bp > 2814.5),

0.0055174931711365043235 * (round_Degree > 3.5 and

round_Closeness > 0.3149999976158142 and

round_StdDev_Transcript_length > 626.7237548828125),

0.14400903577366730435 * (not Group == u'NDNE' and

round_Degree > 3.5 and

round_Closeness <= 0.3149999976158142 and

round_Gene_Length_bp > 41501.0),

0.004987097945538400412 * (round_Blomen_KBM7 > -0.5154723525047302 and

round_missense_Z <= 2.6123709678649902 and

round_StdDev_Transcript_length > 166.57864379882812 and

round_Exon_Count <= 190.5),

-0.010315157710437320229 * (round_Degree <= 18.5 and

0.659500002861023 < round_LofTool <= 0.9602000117301941),

0.0073511514944097225768 * (round_Degree <= 35.5 and

round_Degree_mi <= 0.5 and

round_LofTool <= 0.874500036239624 and

round_StdDev_Transcript_length > 714.5440063476562),

-0.010425093114536482589 * (round_End > 88799552.0 and

round_dN_dS_Chimp <= 0.5950000286102295 and

round_Phi > 0.9999969005584717 and

round_StdDev_Transcript_length > 2430.197265625),

0.018201114582529324265 * (not Group == u'CM' and

Group == u'MNC' and

round_Blomen_KBM7 > -0.5147985219955444 and

round_Gene_Length_bp > 37999.5),

0.0099794866530598415333 * (not Group == u'CM' and

not Group == u'MNC' and

round_Tajima__s_D_regulatory <= 0.4104999899864197 and

round_StdDev_Transcript_length <= 1954.456787109375),

-0.020922796241062330269 * (round_Degree_mi > 0.5 and

round_Average_Transcript_length <= 2606.02392578125),

0.10794282229118463967 * (round_missense_Z <= 3.2976694107055664 and

round_Average_Transcript_length > 2056.5712890625 and

round_Exon_Count > 240.5),

0.023013888862715946998 * (not Group == u'CM' and

round_Closeness > 0.3149999976158142 and

round_Blomen_KBM7 <= -0.5403047800064087 and

round_Transcript_count > 10.5),

-0.18056075313492864209 * (not Group == u'NDNE' and

round_Degree <= 3.5),

-0.0039957720980683267623 * (round_Closeness <= 0.3149999976158142 and

round_dN_dS_Chimp <= 0.5950000286102295 and

round_LofTool <= 0.874500036239624 and

round_Exon_Count <= 171.5),

-0.56961177766960269242 * (Group == u'NDNE' and

round_Degree <= 3.5 and

round_StdDev_Transcript_length <= 714.263427734375),

-0.010926747552256092094 * (round_Degree_mi <= 0.5 and

round_Closeness <= 0.32499998807907104 and

round_LofTool <= 0.9435499906539917 and

round_Gene_Length_bp > 54517.0),

0.0046976857155946452269 * (not Group == u'NDNE' and

round_Degree > 12.5 and

round_Gene_Length_bp <= 53079.0),

0.021939335798790569193 * (round_Phi),

-0.022286463855471616569 * (round_Tajima__s_D_regulatory > 0.4235000014305115 and

round_Blomen_KBM7 > -0.21081802248954773 and

round_s_het > 0.015080630779266357 and

round_StdDev_Transcript_length > 580.992431640625),

0.0027537702678576865545 * (round_Tajima__s_D_regulatory <= 0.4104999899864197 and

round_Phi > 0.0002437000221107155 and

round_missense_Z <= 3.308867931365967),

-0.0027288874703135009708 * (round_End > 127204736.0 and

round_Tajima__s_D_regulatory <= 0.4599999785423279 and

round_Transcript_count <= 10.5 and

round_Gene_Length_bp > 2814.5),

0.08109139290614296447 * (round_missense_Z_mi),

0.014012303940575399075 * (round_missense_Z <= 3.2976694107055664 and

round_LofTool > 0.9603500366210938),

0.052541363893365403137 * (not Group == u'NDNE' and

round_Degree_mi <= 0.5 and

round_Closeness > 0.3149999976158142 and

round_Average_Transcript_length > 2570.535888671875),

0.1551699454009141943 * (round_Tajima__s_D_regulatory <= 0.4235000014305115 and

round_Blomen_KBM7 <= -0.2521226406097412 and

round_s_het > 0.015603477135300636 and

round_Transcript_count <= 21.5),

-0.0080792105837217097208 * (round_Tajima__s_D_regulatory > 0.4104999899864197 and

round_Average_Transcript_length <= 1876.067626953125),

0.021504577862045057279 * (round_End > 125209176.0 and

round_Degree > 12.5 and

round_dN_dS_Chimp_mi <= 0.5),

-0.015295315530416180028 * (round_Degree <= 56.5 and

round_Closeness > 0.2549999952316284 and

round_s_het <= 0.031048648059368134 and

round_Gene_Length_bp > 41216.0),

-0.0078502999183946500783 * (round_Degree <= 3.5 and

round_s_het > 0.02518850564956665 and

round_Transcript_count <= 15.5),

0.024239284046736895434 * (not Group == u'MNC' and

round_dN_dS_Chimp <= 0.5950000286102295 and

round_missense_Z <= 3.2976694107055664 and

round_Average_Transcript_length <= 2056.5712890625),

0.03864183015657941811 * (round_Degree > 4.5 and

round_Blomen_KBM7 <= -0.15796872973442078 and

round_StdDev_Transcript_length > 1227.5733642578125),

0.012003822160604299754 * (not Group == u'MNC' and

round_missense_Z > 3.2976694107055664),

0.01265667266342007137 * (3.5 < round_Degree <= 70.0 and

round_Phi > 0.12447576969861984 and

round_Exon_Count <= 156.5),

-0.025979339010908503865 * (Group == u'NDNE' and

round_Closeness > 0.2549999952316284 and

round_Phi > 0.0014898625668138266 and

round_StdDev_Transcript_length > 636.7213134765625),

0.088736011639851314348 * (not Group == u'CM' and

round_End > 110010712.0 and

round_Degree_mi <= 0.5 and

round_missense_Z <= 3.996763229370117),

-0.042684495370579833562 * (round_Tajima__s_D_regulatory <= 1.4184999465942383 and

round_Transcript_count <= 2.5 and

round_Gene_Length_bp > 9942.0 and

round_Average_Transcript_length > 2037.56787109375),

0.010718660893401628365 * (round_dN_dS_Chimp_mi <= 0.5 and

round_Blomen_KBM7 <= -0.5181520581245422 and

round_Transcript_count <= 26.5 and

round_StdDev_Transcript_length > 169.24288940429688),

-0.0044768437453202277257 * (round_Closeness > 0.2549999952316284 and

round_Blomen_KBM7_mi <= 0.5 and

round_s_het_mi <= 0.5 and

round_StdDev_Transcript_length <= 2625.484375),

0.012631834697413829582 * (2814.5 < round_Gene_Length_bp <= 9338.5 and

round_StdDev_Transcript_length > 580.992431640625),

0.026831902960513024509 * (round_Closeness > 0.33500000834465027 and

3.2976694107055664 < round_missense_Z <= 4.050085067749023),

0.0033918172563335005285 * (round_Blomen_KBM7 > -0.5146373510360718 and

round_Transcript_count > 10.5 and

round_Average_Transcript_length > 2037.5650634765625),

0.0070956564876167357164 * (round_End <= 124517040.0 and

round_Tajima__s_D_regulatory > 0.5564999580383301 and

round_Blomen_KBM7 <= -0.2610846161842346 and

round_Gene_Length_bp > 2814.5),

0.05358215945498377708 * (round_End <= 124883040.0 and

-1.2874999046325684 < round_Tajima__s_D_regulatory <= 1.4165000915527344 and

round_Transcript_count <= 20.5),

0.010564605410865832158 * (round_Degree > 45.0 and

round_dN_dS_Chimp_mi > 0.5 and

round_Transcript_count <= 26.5 and

round_Gene_Length_bp > 2402.0),

0.064211045541165565065 * (round_Degree <= 4.5 and

round_Degree_mi <= 0.5 and

round_StdDev_Transcript_length > 1301.4395751953125),

-0.099504943567778156299 * (Group == u'NDNE' and

round_Degree > 3.5 and

round_missense_Z <= 2.3079466819763184),

0.16847444975170958181 * (round_Closeness <= 0.3149999976158142 and

2.642360210418701 < round_missense_Z <= 4.0604472160339355),

0.04243059279242206161 * (round_Closeness <= 0.3050000071525574 and

round_Blomen_KBM7 > -0.5158457159996033 and

round_Gene_Length_bp <= 53140.5),

0.047538526872885802921 * (not Group == u'NDNE' and

round_Closeness > 0.2549999952316284 and

round_Phi > 0.8754478693008423),

0.01377767556161369443 * (round_Tajima__s_D_regulatory <= 1.4184999465942383 and

round_Transcript_count > 2.5 and

round_Gene_Length_bp > 9942.0 and

round_Average_Transcript_length > 2037.56787109375),

-0.01544246978038381346 * (Group == u'NDNE' and

round_Degree > 3.5),

-0.031626465358424796226 * (round_Degree_mi > 0.5 and

round_Phi <= 0.1322648823261261),

0.023920451949571132355 * (not Group == u'NDNE' and

round_Degree > 3.5 and

round_LofTool > 0.9829000234603882),

0.026861723913622247845 * (round_Closeness > 0.3149999976158142 and

round_Tajima__s_D_regulatory > 0.4104999899864197),

0.0036581146817508628649 * (round_End > 100419720.0 and

round_Tajima__s_D_regulatory <= -0.234499990940094 and

round_missense_Z <= 2.6417436599731445),

-0.037779318023490326972 * (round_Closeness > 0.3149999976158142 and

round_Phi <= 3.313508932478726e-05 and

round_LofTool > 0.04450000077486038),

0.021565100631107423507 * (round_missense_Z <= 3.2976694107055664 and

2821.0 < round_Gene_Length_bp <= 9941.5 and

round_Average_Transcript_length <= 2071.857421875),

-0.018406415026041244437 * (round_Closeness <= 0.3149999976158142 and

round_Phi <= 0.0015477617271244526),

0.044321413351531856184 * (round_End > 100419720.0 and

round_Tajima__s_D_regulatory <= 0.5145000219345093 and

round_missense_Z <= 3.996763229370117 and

round_StdDev_Transcript_length > 166.57864379882812),

-0.040937321521144612313 * (0.3050000071525574 < round_Closeness <= 0.3149999976158142 and

round_Tajima__s_D_regulatory > 0.4104999899864197),

-0.01205681028131431326 * (round_Degree > 4.5 and

round_Gene_Length_bp <= 39298.0),

0.01376055986142680175 * (round_dN_dS_Chimp <= 0.02499999850988388 and

round_dN_dS_Chimp_mi <= 0.5 and

round_LofTool <= 0.9922449588775635 and

round_Average_Transcript_length > 2037.5650634765625),

0.0012393689458476812339 * (round_dN_dS_Chimp <= 0.5950000286102295 and

round_missense_Z <= 2.571293354034424 and

168.6014404296875 < round_StdDev_Transcript_length <= 1450.0491943359375),

-0.0077058749532958803127 * (round_Tajima__s_D_regulatory <= 1.0544999837875366 and

round_Blomen_KBM7 > -0.5153281092643738 and

round_missense_Z_mi <= 0.5 and

round_Transcript_count <= 20.5),

0.035821139812792640589 * (round_Tajima__s_D_regulatory <= -1.2855000495910645 and

round_LofTool > 0.8144999742507935 and

round_StdDev_Transcript_length > 3607.19140625),

0.011287896175946629182 * (Group == u'NDNE' and

round_Closeness > 0.2549999952316284 and

round_Phi > 0.8754478693008423),

-0.086681950407499250288 * (not Group == u'CM' and

not Group == u'MNC' and

round_Degree <= 60.5 and

round_missense_Z > 4.031624794006348),

-0.055560469246041271907 * (round_Phi <= 0.12447576969861984 and

round_StdDev_Transcript_length <= 1098.5848388671875),

0.056832869755874752815 * (round_End <= 100419720.0 and

round_Blomen_KBM7 <= -0.11097116768360138 and

round_missense_Z > 3.2974047660827637),

0.0042022538063266699077 * (round_Closeness <= 0.7749999761581421 and

round_dN_dS_Chimp <= 0.5950000286102295 and

round_Tajima__s_D_regulatory <= 0.34049999713897705 and

round_Blomen_KBM7 > -0.5153244733810425),

0.010232660118857737214 * (not Group == u'NDNE' and

round_End <= 100419720.0 and

round_Phi <= 0.998741626739502 and

round_s_het > 0.017704255878925323),

-0.014904888524450881845 * (round_Closeness <= 0.3149999976158142 and

round_dN_dS_Chimp <= 0.5950000286102295 and

round_Blomen_KBM7 > -0.514461874961853 and

round_StdDev_Transcript_length <= 1944.22802734375),

-0.12345581397405855362 * (round_Closeness <= 0.3149999976158142 and

round_Phi <= 3.313508932478726e-05 and

round_LofTool > 0.04450000077486038),

-0.0082097542006331434422 * (Group == u'NDNE' and

round_End <= 100419720.0 and

round_Closeness <= 0.3149999976158142 and

round_Blomen_KBM7 <= -0.25102293491363525),

0.0066597396499948708845 * (round_End <= 100419720.0 and

round_Degree <= 12.5 and

round_dN_dS_Chimp_mi <= 0.5 and

round_Exon_Count <= 221.5),

-0.026228336463867144013 * (not Group == u'CM' and

round_End > 127204736.0 and

round_Phi > 0.9981463551521301 and

round_s_het > 0.1433388888835907),

0.0072832761262565624827 * (round_End > 100419720.0 and

round_missense_Z > 2.6417436599731445),

-0.018198365192935522794 * (4.5 < round_Degree <= 31.5 and

round_Phi <= 0.919446587562561 and

round_Gene_Length_bp > 39298.0),

-0.0014469117791570695365 * (0.659500002861023 < round_LofTool <= 0.9921150207519531 and

round_StdDev_Transcript_length > 589.7366333007812 and

round_Exon_Count <= 174.5),

0.04649175361763767389 * (round_LofTool > 0.6634999513626099 and

round_Exon_Count > 156.5),

-0.040594818357408524179 * (Group == u'NDNE' and

round_s_het > 0.016313210129737854 and

round_Gene_Length_bp <= 38001.5),

-0.067706065723669231482 * (round_dN_dS_Chimp_mi > 0.5 and

round_Blomen_KBM7 > -0.5146373510360718 and

round_Transcript_count <= 20.5 and

round_StdDev_Transcript_length <= 2388.5126953125),

0.019417071016638930842 * (round_End <= 120516032.0 and

round_Closeness <= 0.33500000834465027 and

round_Tajima__s_D_regulatory <= 0.4104999899864197 and

round_Exon_Count > 97.5),

0.022064341778839532265 * (round_Transcript_count > 20.5 and

round_StdDev_Transcript_length <= 2418.21435546875),

0.0044133194936464073543 * (not Group == u'NDNE' and

round_Tajima__s_D_regulatory <= 0.5570000410079956 and

round_LofTool > 0.8105000257492065),

-0.14398676711490873692 * (not Group == u'MNC' and

round_Tajima__s_D_regulatory <= -0.4115000069141388 and

8050.5 < round_Gene_Length_bp <= 53048.5),

0.040024524551637907788 * (not Group == u'NDNE' and

round_Closeness > 0.2549999952316284 and

round_Transcript_count > 15.5),

-0.015835246306718037124 * (round_Closeness <= 0.3149999976158142 and

round_dN_dS_Chimp <= 0.5950000286102295 and

round_Blomen_KBM7 <= -0.514461874961853 and

round_StdDev_Transcript_length <= 1944.22802734375),

0.010716122739572278219 * (round_Degree > 60.5 and

round_Blomen_KBM7 > -0.5153281092643738),

-0.0037798883504378804482 * (round_dN_dS_Chimp <= 0.5950000286102295 and

round_missense_Z > 4.052361965179443 and

round_LofTool <= 0.9922449588775635 and

round_Exon_Count <= 108.5),

-0.0015681404390947323475 * (round_dN_dS_Chimp_mi > 0.5 and

round_Average_Transcript_length <= 2037.5650634765625),

0.038785018347274782813 * (not Group == u'NDNE' and

round_Degree > 3.5 and

round_LofTool <= 0.6514999866485596),

-0.0039977514872525012762 * (Group == u'NDNE' and

round_dN_dS_Chimp_mi <= 0.5 and

round_Transcript_count <= 26.5 and

round_StdDev_Transcript_length <= 2388.5126953125),

0.038112358760383956147 * (Group == u'NDNE' and

round_Degree_mi > 0.5),

0.026371943455055456978 * (not Group == u'MNC' and

round_Phi > 3.313508932478726e-05 and

3.2976694107055664 < round_missense_Z <= 4.052361965179443),

0.049444141026331135669 * (not Group == u'CM' and

round_Tajima__s_D_regulatory > 0.4235000014305115 and

round_s_het > 0.014262920245528221),

0.013937161271230649046 * (not Group == u'NDNE' and

round_Closeness > 0.3149999976158142 and

round_missense_Z <= 1.43977952003479),

-0.020864630641113317278 * (round_Degree <= 12.5 and

round_missense_Z > 4.052361965179443 and

round_StdDev_Transcript_length <= 2616.1044921875),

0.010891575439352343263 * (not Group == u'CM' and

round_Phi > 3.318415838293731e-05 and

round_Blomen_KBM7 > -0.5403047800064087 and

round_Transcript_count > 10.5),

-0.020197315811600891067 * (round_Tajima__s_D_regulatory > -1.2874999046325684 and

round_Blomen_KBM7 > -0.12487166374921799 and

round_s_het > 0.02633928880095482 and

round_Exon_Count <= 53.0),

-0.025658439979292017169 * (round_Closeness <= 0.3149999976158142 and

round_s_het <= 0.025150161236524582 and

round_Average_Transcript_length <= 2057.064453125),

-0.042894291214758038799 * (round_Closeness <= 0.3149999976158142 and

round_dN_dS_Chimp_mi <= 0.5 and

round_Gene_Length_bp > 9941.5 and

round_Average_Transcript_length <= 2037.5650634765625),

0.0114569281039716038 * (not Group == u'NDNE' and

round_Closeness <= 0.32499998807907104 and

round_Tajima__s_D_regulatory <= 0.5564999580383301 and

round_Gene_Length_bp <= 47758.5),

0.067766934758570276931 * (not Group == u'NDNE' and

round_Closeness > 0.2549999952316284 and

round_LofTool > 0.9787000417709351),

0.042775949189413867146 * (not Group == u'CM' and

round_End <= 127204736.0 and

round_Degree > 61.5),

-0.033595605379461213058 * (round_missense_Z > 4.052361965179443 and

round_LofTool <= 0.9807000160217285),

0.017127149696538990914 * (round_Gene_Length_bp > 37833.5 and

round_StdDev_Transcript_length <= 1956.23095703125),

0.011030940167582190675 * (round_Degree <= 12.5 and

round_Blomen_KBM7 <= -0.1940726488828659 and

round_s_het > 0.025150161236524582 and

round_Transcript_count <= 20.5),

-0.25855416246864998397 * (round_Phi <= 0.00015248148702085018 and

round_LofTool <= 0.6634999513626099 and

round_Exon_Count <= 157.5),

0.018976143304323109251 * (round_End > 100419720.0 and

round_dN_dS_Chimp_mi <= 0.5 and

round_Blomen_KBM7 > -0.5072991847991943 and

round_LofTool <= 0.994350016117096),

0.0010472512424781869628 * (round_LofTool <= 0.6655000448226929 and

round_s_het > 0.01709270477294922 and

round_Exon_Count <= 87.5),

-0.018696452857061698211 * (not Group == u'CM' and

round_Degree <= 64.0 and

round_dN_dS_Chimp_mi > 0.5 and

round_StdDev_Transcript_length <= 1942.072509765625),

0.011962027921206701275 * (not Group == u'NDNE' and

12.5 < round_Degree <= 63.5 and

round_Degree_mi <= 0.5),

0.15322978417035446053 * (round_Degree <= 4.5 and

round_LofTool <= 0.9868500232696533 and

round_StdDev_Transcript_length > 1049.45654296875),

0.046437531520907876503 * (not Group == u'NDNE' and

round_Closeness > 0.2549999952316284 and

round_LofTool <= 0.9787000417709351 and

round_StdDev_Transcript_length > 1005.2410888671875),

-0.039805408645227642606 * (round_Degree <= 35.5 and

round_Blomen_KBM7 > -0.2521226406097412 and

round_s_het <= 0.01751864142715931 and

round_Gene_Length_bp > 2814.5),

-0.070420047677563976651 * (Group == u'NDNE' and

round_Degree > 3.5 and

round_missense_Z > 2.3079466819763184),

0.0025535846861198395648 * (Group == u'NDNE' and

round_Degree <= 35.5 and

round_Tajima__s_D_regulatory <= 1.0544999837875366 and

round_missense_Z <= 4.052361965179443),

0.092506701425168813557 * (round_Tajima__s_D_regulatory <= 0.4235000014305115 and

round_Blomen_KBM7 > -0.2521226406097412),

0.028016343518460877504 * (round_Degree > 4.5 and

round_missense_Z > 1.43977952003479 and

round_Transcript_count <= 21.5),

0.013589678385553336654 * (round_End <= 100419720.0 and

round_Degree > 12.5 and

round_LofTool <= 0.812000036239624 and

round_Exon_Count <= 221.5),

-0.010867009106563865761 * (round_Degree <= 60.5 and

round_Tajima__s_D_regulatory > -0.7795000076293945 and

round_Blomen_KBM7 > -0.5153281092643738 and

round_Exon_Count > 154.5),

-0.0036726609659116712936 * (round_Degree_mi <= 0.5 and

round_missense_Z <= 2.552614450454712 and

round_LofTool > 0.6644999980926514 and

round_Average_Transcript_length <= 2570.535888671875),

0.013832036762706318919 * (round_Closeness <= 0.7749999761581421 and

round_dN_dS_Chimp <= 0.5950000286102295 and

round_Tajima__s_D_regulatory <= 0.34049999713897705 and

round_Blomen_KBM7 <= -0.5153244733810425),

0.053044796037296365609 * (round_Degree > 12.5 and

round_LofTool <= 0.9921150207519531 and

round_s_het > 0.025150161236524582 and

round_Exon_Count > 224.5),

-0.013287956853066012 * (not Group == u'MNC' and

round_dN_dS_Chimp_mi <= 0.5 and

round_Blomen_KBM7 > -0.5146373510360718 and

round_Gene_Length_bp <= 37999.5),

0.10324470994342001273 * (round_LofTool > 0.9921150207519531),

0.0057921807143307855667 * (not Group == u'CM' and

round_Closeness <= 0.3149999976158142 and

round_Blomen_KBM7 <= -0.5403047800064087 and

round_Transcript_count > 10.5),

0.092157718501607158168 * (round_Degree_mi <= 0.5 and

round_Blomen_KBM7 <= -0.15531033277511597 and

round_missense_Z > 2.721888303756714 and

round_Exon_Count > 262.0),

-0.1305724926220721005 * (round_Degree_mi > 0.5 and

round_StdDev_Transcript_length <= 1060.87158203125),

0.016193134371026433188 * (round_Degree <= 35.5 and

round_Degree_mi <= 0.5 and

round_LofTool <= 0.874500036239624 and

round_StdDev_Transcript_length <= 714.5440063476562),

0.035774440858628839268 * (Group == u'CM' and

round_Tajima__s_D_regulatory > 0.4165000021457672),

-0.0069014727149561538172 * (round_missense_Z <= 4.054958820343018 and

round_Transcript_count <= 10.5 and

round_Gene_Length_bp > 10637.0 and

round_Average_Transcript_length > 2037.5650634765625),

-0.026771080458371804972 * (round_Closeness <= 0.2549999952316284 and

round_Phi > 0.8162848949432373 and

round_LofTool <= 0.9833999872207642),

-0.022253783562079281627 * (round_Closeness <= 0.26499998569488525 and

round_LofTool <= 0.8105000257492065 and

round_Gene_Length_bp <= 37966.5),

-0.0024688924457943600688 * (not Group == u'MNC' and

-1.2874999046325684 < round_Tajima__s_D_regulatory <= 1.2934999465942383 and

round_Blomen_KBM7 > -0.4999815821647644),

0.042899930949984906026 * (not Group == u'NDNE' and

round_missense_Z > 1.43977952003479 and

round_StdDev_Transcript_length > 586.8510131835938 and

round_Exon_Count <= 229.5),

0.017509022546737609133 * (round_End > 100419720.0),

-0.066444044833209647827 * (round_Tajima__s_D_regulatory > -1.2874999046325684 and

round_Blomen_KBM7 <= -0.12487166374921799 and

3.134337902069092 < round_missense_Z <= 3.2974047660827637),

-0.00219063763450522489 * (not Group == u'NDNE' and

round_Degree <= 4.5 and

round_Phi > 0.8875079154968262),

0.11064953698387736125 * (Group == u'CM' and

round_End > 110010712.0 and

round_Degree_mi <= 0.5),

-0.059270604135849759564 * (Group == u'NDNE' and

round_missense_Z <= 2.7258143424987793 and

round_Exon_Count <= 171.5),

-0.018291629266993809227 * (round_Degree <= 12.5 and

round_Blomen_KBM7 > -0.12487166374921799),

0.035226494727457799416 * (round_Phi > 0.12447576969861984 and

93.5 < round_Exon_Count <= 265.5),

0.050722711654871383002 * (round_Degree > 12.5 and

round_Closeness > 0.33500000834465027 and

round_Tajima__s_D_regulatory <= 0.4235000014305115 and

round_LofTool <= 0.9922800064086914),

-0.051814515969269794859 * (round_Closeness <= 0.3149999976158142 and

round_Phi <= 0.12447576969861984 and

round_Exon_Count <= 83.5),

-0.004095557220962523122 * (round_Degree > 12.5 and

round_dN_dS_Chimp <= 0.5950000286102295 and

round_missense_Z > 4.031624794006348 and

round_LofTool <= 0.9499499797821045),

-0.061167712425044540314 * (round_Degree_mi > 0.5 and

round_Gene_Length_bp <= 2510.5),

-0.0182849435306143282 * (Group == u'MNC' and

round_missense_Z <= 3.2976694107055664 and

round_StdDev_Transcript_length <= 987.5745849609375),

-0.32389234107127440332 * (Group == u'NDNE' and

round_Closeness <= 0.2549999952316284 and

round_Phi <= 0.14362311363220215),

-0.14541641777476235764 * (Group == u'MNC' and

round_Gene_Length_bp <= 53048.5),

0.0087320822755329839671 * (round_Degree <= 12.5 and

round_Degree_mi <= 0.5 and

round_missense_Z > 2.120723009109497 and

round_StdDev_Transcript_length > 1011.6593627929688),

0.017852506892799022836 * (round_Closeness > 0.2549999952316284 and

3.334120750427246 < round_missense_Z <= 4.052361965179443 and

round_s_het > 0.014458265155553818),

-0.038423746283818283054 * (round_Degree <= 3.5 and

round_Degree_mi > 0.5 and

round_s_het <= 0.023678744211792946),

-0.004412961621996456911 * (not Group == u'CM' and

round_Tajima__s_D_regulatory <= 0.9564999938011169 and

round_Blomen_KBM7 > -0.5147985219955444 and

round_Gene_Length_bp <= 37999.5),

0.050996412382406750008 * (not Group == u'MNC' and

round_missense_Z <= 2.642360210418701 and

round_s_het > 0.014316117390990257 and

round_Exon_Count <= 165.5),

0.017338694268998987996 * (not Group == u'NDNE' and

round_Degree > 3.5 and

round_Closeness > 0.3149999976158142),

-0.023156005411269445921 * (not Group == u'CM' and

round_End <= 127204736.0 and

round_Degree <= 61.5 and

round_missense_Z <= 4.031624794006348),

0.0028308618694010165111 * (round_dN_dS_Chimp_mi <= 0.5 and

round_Blomen_KBM7 > -0.5146373510360718 and

round_LofTool <= 0.9922449588775635 and

round_Transcript_count <= 20.5),

-0.040546423472875826877 * (Group == u'NDNE' and

round_missense_Z <= 2.7258143424987793 and

round_Exon_Count > 171.5),

-0.015113303914765858355 * (not Group == u'CM' and

round_End > 127204736.0 and

round_Phi > 0.9974162578582764 and

round_StdDev_Transcript_length <= 1345.761474609375),

-0.10222460154756490835 * (round_Closeness <= 0.2549999952316284 and

round_Phi > 0.0002437000221107155 and

round_missense_Z <= 4.0604472160339355 and

round_StdDev_Transcript_length > 615.416259765625),

0.012362969768704433135 * (not Group == u'MNC' and

round_Degree > 34.5 and

round_Degree_mi <= 0.5 and

round_missense_Z <= 4.0604472160339355),

-0.0064503231243915092399 * (round_Tajima__s_D_regulatory <= 0.43549999594688416 and

round_missense_Z <= 3.2976694107055664 and

round_LofTool <= 0.9603500366210938 and

round_Exon_Count <= 164.5),

-0.0022205334822635804277 * (not Group == u'CM' and

round_Tajima__s_D_regulatory > -1.2894999980926514 and

round_Blomen_KBM7 <= -0.5147985219955444 and

round_Transcript_count <= 26.5),

-0.25240843520512323828 * (round_Phi > 0.00015248148702085018 and

round_Blomen_KBM7 > -0.15492522716522217 and

round_LofTool <= 0.6634999513626099 and

round_Exon_Count <= 157.5),

0.0095476722071454571406 * (not Group == u'NDNE' and

round_Closeness <= 0.32499998807907104 and

round_Tajima__s_D_regulatory <= 0.5564999580383301 and

round_Gene_Length_bp > 47758.5),

0.025785796597482577019 * (not Group == u'NDNE' and

round_Degree > 3.5 and

round_LofTool > 0.9787000417709351),

0.1378685779507847764 * (not Group == u'NDNE' and

round_Closeness > 0.3149999976158142 and

round_Phi > 0.8768634796142578),

0.0024299681294777109031 * (round_End <= 128392288.0 and

round_Gene_Length_bp > 9338.5 and

round_StdDev_Transcript_length > 1956.23095703125),

0.043814273445441413724 * (round_Degree_mi <= 0.5 and

round_Blomen_KBM7 <= -0.14571721851825714 and

round_LofTool > 0.9435499906539917),

0.0033544123090482034534 * (round_missense_Z <= 2.6123709678649902 and

round_missense_Z_mi <= 0.5 and

round_Gene_Length_bp > 9941.5 and

round_StdDev_Transcript_length > 166.57949829101562),

0.027254350066886999515 * (round_Closeness > 0.33500000834465027 and

round_Tajima__s_D_regulatory <= 0.5564999580383301),

-0.0021195491504552806984 * (round_Degree <= 35.5 and

round_Gene_Length_bp > 9906.5 and

round_StdDev_Transcript_length > 166.57864379882812 and

round_Average_Transcript_length <= 1956.6083984375),

0.048251921050298546279 * (round_Degree > 12.5 and

round_Degree_mi <= 0.5 and

round_Closeness <= 0.3149999976158142 and

round_StdDev_Transcript_length > 1285.35791015625),

0.022409297933030165179 * (round_Degree > 3.5 and

round_Closeness <= 0.3149999976158142 and

round_LofTool <= 0.6775000095367432 and

round_Exon_Count <= 151.5),

0.045353884427582646932 * (round_Gene_Length_bp <= 9906.5 and

round_StdDev_Transcript_length > 166.57864379882812 and

round_Average_Transcript_length > 1577.136474609375),

-0.012720933738028348745 * (round_Phi <= 0.12447576969861984 and

round_StdDev_Transcript_length <= 1452.739013671875),

0.0020671485816952722345 * (round_Tajima__s_D_regulatory <= 1.0544999837875366 and

round_Phi <= 0.9990512132644653 and

round_Blomen_KBM7 > -0.5153281092643738 and

round_Transcript_count > 20.5),

-0.0072592442110520818271 * (round_Degree_mi > 0.5 and

round_Average_Transcript_length <= 2170.1923828125),

0.0016667596308927788533 * (3.308867931365967 < round_missense_Z <= 4.052361965179443 and

round_Exon_Count > 97.5),

0.01867224397834999633 * (round_Blomen_KBM7 <= -0.2516775131225586 and

3.308867931365967 < round_missense_Z <= 4.0613203048706055 and

round_s_het > 0.015855055302381516),

0.061536203645746870294 * (round_dN_dS_Chimp <= 0.5950000286102295 and

round_LofTool > 0.9922449588775635),

-0.01865222650192009321 * (not Group == u'CM' and

round_dN_dS_Chimp_mi <= 0.5 and

round_Blomen_KBM7_mi > 0.5 and

round_StdDev_Transcript_length <= 2769.32568359375),

-0.031063061425573603586 * (round_dN_dS_Chimp_mi <= 0.5 and

round_StdDev_Transcript_length <= 168.6014404296875),

0.016728971448681104889 * (round_Degree > 10.5 and

round_Degree_mi <= 0.5 and

round_LofTool <= 0.9311000108718872 and

round_s_het > 0.025092266499996185),

-0.30582887886172710479 * (round_Degree <= 4.5 and

round_Phi <= 0.14253592491149902 and

round_StdDev_Transcript_length > 616.5709228515625),

0.017548642979559044702 * (round_End <= 100419720.0 and

round_missense_Z <= 4.031624794006348 and

round_StdDev_Transcript_length > 166.57864379882812 and

round_Exon_Count <= 221.5),

0.033533794559069317331 * (round_End <= 100419720.0 and

round_Degree > 12.5 and

round_LofTool > 0.812000036239624 and

round_Exon_Count <= 221.5),

0.0065947414569974410758 * (not Group == u'MNC' and

round_End > 100419720.0 and

round_Tajima__s_D_regulatory > -1.2855000495910645 and

round_StdDev_Transcript_length <= 635.1746826171875),

-0.0056566784579737171626 * (round_Degree > 4.5 and

round_Closeness <= 0.3149999976158142 and

round_Transcript_count > 24.5 and

round_StdDev_Transcript_length > 897.559326171875),

0.081239321752520798903 * (round_Tajima__s_D_regulatory > -1.2874999046325684 and

3.2976694107055664 < round_missense_Z <= 3.8540451526641846),

0.030930760440212312634 * (round_Degree > 35.5 and

round_Degree_mi <= 0.5 and

round_Blomen_KBM7 <= -0.1796257197856903 and

round_LofTool <= 0.874500036239624),

-0.0074955400533961748233 * (round_Closeness > 0.2549999952316284 and

round_Phi > 0.0015477617271244526 and

round_StdDev_Transcript_length <= 2427.245361328125 and

round_Exon_Count <= 96.5),

0.0060206295646320932488 * (round_dN_dS_Chimp_mi <= 0.5 and

round_s_het > 0.01754925772547722 and

round_Transcript_count <= 26.5 and

round_StdDev_Transcript_length > 169.24288940429688),

-0.022479192865490577047 * (Group == u'NDNE' and

round_Degree <= 8.5 and

round_Phi <= 0.18718643486499786 and

round_StdDev_Transcript_length > 714.5440063476562),

-0.004902590801046578968 * (not Group == u'MNC' and

round_s_het <= 0.015855055302381516),

-0.017831132034095014544 * (Group == u'NDNE' and

round_End <= 100419720.0 and

round_Closeness <= 0.3149999976158142 and

round_Blomen_KBM7 > -0.25102293491363525),

0.013623595360248288294 * (round_Closeness > 0.2549999952316284 and

round_dN_dS_Chimp <= 0.5950000286102295 and

round_StdDev_Transcript_length <= 2430.197265625 and

round_Exon_Count <= 246.0),

0.018865549838809763522 * (round_dN_dS_Chimp_mi <= 0.5 and

round_missense_Z <= 3.2976694107055664 and

round_StdDev_Transcript_length > 166.57864379882812 and

round_Average_Transcript_length <= 2071.857421875),

0.014732213904117162293 * (round_Degree > 12.5 and

round_dN_dS_Chimp_mi <= 0.5 and

round_s_het > 0.015838049352169037 and

round_StdDev_Transcript_length > 168.6014404296875),

-0.016714848080446102069 * (not Group == u'CM' and

round_Tajima__s_D_regulatory > 0.9564999938011169 and

round_Blomen_KBM7 > -0.5147985219955444 and

round_Gene_Length_bp <= 37999.5),

-0.015963145907007737778 * (round_StdDev_Transcript_length <= 590.2589111328125),

-0.0060448945213366633844 * (Group == u'NDNE' and

round_Degree_mi <= 0.5 and

round_s_het > 0.02767527475953102),

0.027704609106936072677 * (not Group == u'NDNE' and

round_missense_Z <= 1.43977952003479 and

round_StdDev_Transcript_length > 586.8510131835938 and

round_Exon_Count <= 229.5),

-0.051098898316382507234 * (round_Closeness > 0.2549999952316284 and

3.8498473167419434 < round_missense_Z <= 4.052361965179443),

-0.058386046266751971678 * (round_Blomen_KBM7 > -0.1469864696264267),

0.0060639684811802183756 * (round_Closeness <= 0.35500001907348633 and

round_dN_dS_Chimp <= 0.5849999785423279 and

round_missense_Z <= 4.052361965179443 and

round_Gene_Length_bp > 2814.5),

0.05233613286777938356 * (Group == u'NDNE' and

round_Closeness <= 0.2549999952316284 and

round_Phi > 0.1670895218849182),

-0.17848306160053159508 * (round_Degree <= 4.5 and

round_StdDev_Transcript_length <= 1049.45654296875),

-0.00011540571561985079577 * (round_Tajima__s_D_regulatory <= -1.2855000495910645 and

round_Blomen_KBM7 > -0.5154076814651489 and

round_LofTool <= 0.8144999742507935),

-0.010416764352577573272 * (round_missense_Z <= 3.325303077697754 and

round_LofTool <= 0.9922800064086914 and

round_StdDev_Transcript_length > 2377.90625),

-0.05612691108153916586 * (Group == u'NDNE' and

round_missense_Z > 1.1351158618927002 and

round_Transcript_count <= 10.5),

0.090625893742273977427 * (round_Degree > 3.5 and

round_Closeness > 0.3149999976158142 and

round_Gene_Length_bp <= 41501.0),

0.019908066071534915448 * (round_LofTool > 0.9920099973678589 and

round_Exon_Count > 86.5),

-0.014119188513395540888 * (not Group == u'MNC' and

round_Phi <= 0.0015477617271244526),

0.0046295616520464280552 * (round_Degree > 3.5 and

round_Exon_Count > 156.5),

0.017556588763314336793 * (round_Degree <= 12.5 and

round_Blomen_KBM7 <= -0.1114160418510437 and

round_StdDev_Transcript_length <= 2377.90625 and

round_Average_Transcript_length > 2169.3193359375),

0.0187314523846858344 * (Group == u'NDNE' and

round_Degree_mi <= 0.5 and

round_Phi > 0.00028779974672943354 and

round_StdDev_Transcript_length > 1207.362548828125),

0.021330433143297765353 * (round_Degree <= 64.5 and

round_Degree_mi <= 0.5 and

round_Closeness > 0.3149999976158142 and

round_Exon_Count <= 156.5),

0.021452094564182736663 * (round_Tajima__s_D_regulatory > -1.2874999046325684 and

round_Blomen_KBM7 <= -0.12487166374921799 and

round_missense_Z <= 3.134337902069092),

-0.032839560232847293808 * (round_missense_Z > 3.04426908493042 and

round_StdDev_Transcript_length <= 167.33212280273438),

0.012377636161393038711 * (round_End <= 100419720.0 and

round_Blomen_KBM7 <= -0.5154723525047302 and

round_missense_Z <= 3.2974047660827637),

0.00011644535307463677531 * (round_Degree),

0.011337114232236933375 * (round_Degree_mi <= 0.5 and

round_missense_Z > 1.43977952003479 and

round_LofTool <= 0.9886499643325806 and

round_Gene_Length_bp > 39341.0),

-0.012961434512462111784 * (not Group == u'NDNE' and

round_Degree <= 12.5 and

round_Degree_mi <= 0.5 and

round_missense_Z <= 2.3431456089019775),

-0.020474012020399504769 * (round_End <= 120572272.0 and

round_Closeness <= 0.32499998807907104 and

round_Tajima__s_D_regulatory <= 0.4235000014305115 and

round_Gene_Length_bp > 54517.0),

-0.010177296319135669886 * (round_Degree <= 10.5 and

round_Degree_mi <= 0.5 and

round_Phi <= 0.13315337896347046 and

round_LofTool <= 0.9311000108718872),

-0.0047629233659651970187 * (round_Tajima__s_D_regulatory > 0.4104999899864197 and

round_Transcript_count <= 10.5 and

round_Average_Transcript_length > 1876.067626953125),

0.0068520762408969563759 * (not Group == u'NDNE' and

round_Degree > 3.5 and

round_Phi <= 0.919446587562561 and

round_StdDev_Transcript_length <= 1039.699462890625),

-0.028130566254400735798 * (Group == u'NDNE' and

round_Degree <= 4.5 and

round_StdDev_Transcript_length <= 621.470947265625),

-0.024145074195087025404 * (round_Degree <= 12.5 and

round_Degree_mi <= 0.5 and

round_Phi <= 0.12362469732761383 and

round_StdDev_Transcript_length <= 1011.6593627929688),

0.0015991642978274947951 * (round_Degree <= 21.5 and

round_Degree_mi <= 0.5 and

round_Exon_Count > 156.5),

0.088647162920468577929 * (Group == u'NDNE' and

round_Degree > 3.5 and

round_Gene_Length_bp > 41501.0),

0.026665282263039657984 * (round_Degree > 4.5 and

round_missense_Z <= 4.0604472160339355 and

round_Transcript_count <= 20.5 and

round_StdDev_Transcript_length > 1967.8238525390625),

-0.0048445704274170222139 * (round_Closeness <= 0.2549999952316284 and

round_missense_Z <= 4.052361965179443 and

round_Gene_Length_bp <= 53173.0),

-0.014231203632158318309 * (not Group == u'MNC' and

round_Tajima__s_D_regulatory > -1.2874999046325684 and

round_Blomen_KBM7 <= -0.12487166374921799 and

round_Gene_Length_bp <= 343581.5),

-0.018978210144039470153 * (Group == u'NDNE' and

round_Closeness <= 0.2549999952316284 and

round_StdDev_Transcript_length > 636.7213134765625),

0.041662196873145339315 * (round_Degree_mi <= 0.5 and

round_Closeness <= 0.3050000071525574 and

round_missense_Z_mi <= 0.5 and

round_Gene_Length_bp > 2814.5),

0.016988971806798404407 * (not Group == u'MNC' and

round_Degree > 62.5 and

round_Degree_mi <= 0.5 and

round_Blomen_KBM7 <= -0.2516775131225586),

-0.023466563573408403404 * (round_Closeness <= 0.3149999976158142 and

round_dN_dS_Chimp_mi <= 0.5 and

round_Gene_Length_bp <= 9941.5 and

round_Average_Transcript_length <= 2037.5650634765625),

-0.021454619164280947646 * (round_Degree <= 12.5 and

round_missense_Z > 4.052361965179443 and

round_Gene_Length_bp <= 110374.0 and

round_Average_Transcript_length <= 2180.86572265625),

0.022697035856952464672 * (round_End <= 100419720.0 and

round_Exon_Count > 221.5),

0.0081456657325367897576 * (not Group == u'CM' and

not Group == u'MNC' and

round_Blomen_KBM7 > -0.5148604512214661 and

round_Transcript_count > 10.5),

0.029906081927261407571 * (round_missense_Z <= 4.054958820343018 and

round_Transcript_count <= 10.5 and

round_Gene_Length_bp <= 10637.0 and

round_Average_Transcript_length > 2037.5650634765625),

0.047761530637367016761 * (round_End <= 88799552.0 and

round_dN_dS_Chimp <= 0.5950000286102295 and

round_StdDev_Transcript_length > 2430.197265625),

0.038357225265835417916 * (round_Degree > 12.5 and

round_Blomen_KBM7 <= -0.25064072012901306),

0.021452043305304778487 * (not Group == u'CM' and

round_End > 127204736.0 and

round_Phi <= 0.9981463551521301 and

round_Exon_Count > 69.5),

-0.048336036395590260828 * (round_Closeness <= 0.3149999976158142 and

round_Blomen_KBM7 > -0.25219112634658813 and

round_Transcript_count <= 19.5),

0.052244759431406627426 * (round_Tajima__s_D_regulatory <= -1.2874999046325684 and

round_Blomen_KBM7 > -0.41121259331703186),

-0.014142570663065691036 * (round_Blomen_KBM7 <= -0.2516775131225586 and

round_missense_Z > 4.0613203048706055 and

round_s_het > 0.015855055302381516 and

round_Exon_Count <= 224.5),

0.040131506231853936173 * (round_End <= 110010712.0 and

round_Degree > 61.5 and

round_Degree_mi <= 0.5),

-0.052525036398370784918 * (not Group == u'MNC' and

round_Closeness > 0.2549999952316284 and

round_missense_Z <= 3.2976694107055664 and

round_StdDev_Transcript_length > 987.5745849609375),

-0.03444322178607048951 * (round_Degree <= 12.5 and

round_Blomen_KBM7 > -0.1114160418510437),

0.0080778520322766257655 * (round_Phi > 0.9981780648231506 and

round_missense_Z > 2.6123709678649902 and

round_missense_Z_mi <= 0.5 and

round_Average_Transcript_length > 2957.866943359375),

-0.0018453559825937011947 * (not Group == u'MNC' and

round_Phi > 3.313508932478726e-05 and

round_missense_Z <= 3.2976694107055664),

0.088310649006325236954 * (Group == u'CM'),

-0.3321493231824571013 * (round_Degree_mi > 0.5 and

round_StdDev_Transcript_length > 1060.87158203125),

0.089806398288230407378 * (round_Phi <= 3.313508932478726e-05 and

round_missense_Z <= 4.052361965179443 and

round_LofTool <= 0.04450000077486038),

-0.056141049902893203072 * (not Group == u'NDNE' and

round_Closeness <= 0.2549999952316284 and

round_Phi <= 0.9640257358551025),

-0.035048251621686281332 * (round_Degree <= 12.5 and

round_Closeness > 0.3050000071525574 and

round_dN_dS_Chimp_mi <= 0.5 and

round_StdDev_Transcript_length > 168.6014404296875),

0.0049429908706841016106 * (not Group == u'NDNE' and

round_Degree_mi <= 0.5 and

round_Tajima__s_D_regulatory <= 1.0544999837875366 and

round_missense_Z <= 4.052361965179443),

0.025594410691442658068 * (round_End <= 125209176.0 and

round_Degree > 12.5 and

round_dN_dS_Chimp_mi <= 0.5 and

round_StdDev_Transcript_length > 3690.060546875),

0.020970553574886488524 * (round_Closeness > 0.3149999976158142 and

round_Phi > 0.0015477617271244526 and

round_StdDev_Transcript_length > 626.7237548828125 and

round_Exon_Count <= 283.5),

-0.0072708255207664862149 * (round_Degree_mi <= 0.5 and

round_LofTool <= 0.659500002861023 and

round_StdDev_Transcript_length <= 809.498046875),

-0.001003390225380734746 * (round_dN_dS_Chimp > 0.5950000286102295 and

round_Transcript_count <= 26.5 and

round_Average_Transcript_length > 1853.1895751953125),

0.0078267880577386761409 * (not Group == u'CM' and

not Group == u'MNC' and

round_Degree <= 70.0 and

round_LofTool > 0.8695000410079956),

0.30178102419431956926 * (round_Closeness > 0.3149999976158142 and

round_Exon_Count > 224.5),

-0.04163560042881176565 * (round_Closeness <= 0.2549999952316284 and

round_LofTool <= 0.8535000085830688 and

round_StdDev_Transcript_length <= 617.4835815429688),

0.0030910877541061327484 * (round_Phi <= 0.1378774642944336 and

round_LofTool <= 0.659500002861023 and

round_Average_Transcript_length > 2515.3544921875),

0.029649301367085705017 * (round_dN_dS_Chimp_mi > 0.5 and

round_missense_Z <= 3.2976694107055664 and

round_StdDev_Transcript_length > 166.57864379882812),

0.031982851277675909685 * (round_Degree <= 12.5 and

round_Tajima__s_D_regulatory <= 0.4235000014305115 and

round_LofTool <= 0.9922800064086914 and

round_Gene_Length_bp > 37966.5),

-0.017070967066707309207 * (round_dN_dS_Chimp > 0.02499999850988388 and

round_dN_dS_Chimp_mi <= 0.5 and

round_LofTool <= 0.9922449588775635 and

round_Average_Transcript_length > 2037.5650634765625),

0.029724330771197321477 * (round_missense_Z <= 3.1514573097229004 and

round_LofTool <= 0.9922449588775635 and

round_Average_Transcript_length > 2071.857421875),

0.036175061572276949462 * (round_Degree_mi <= 0.5 and

round_LofTool > 0.9886499643325806 and

round_Gene_Length_bp > 39341.0),

0.012711184921391694216 * (round_Phi <= 0.998741626739502 and

round_Gene_Length_bp > 9906.5 and

round_StdDev_Transcript_length > 166.57864379882812 and

round_Average_Transcript_length > 1956.6083984375),

0.036358077235948783879 * (not Group == u'NDNE' and

round_Degree > 3.5 and

round_Closeness <= 0.3149999976158142 and

round_s_het > 0.03478143364191055),

-0.062385456839495603831 * (round_s_het <= 0.016313210129737854 and

round_Gene_Length_bp <= 38001.5),

0.098082437827070073633 * (round_Closeness > 0.3149999976158142 and

round_Transcript_count <= 19.5),

0.011020545071693770359 * (round_Degree > 12.5 and

4.052361965179443 < round_missense_Z <= 7.099285125732422 and

round_Gene_Length_bp > 2814.5),

0.06749773290419343319 * (not Group == u'NDNE' and

round_Degree > 62.5),

-0.01639995939674137107 * (round_Degree > 4.5 and

round_Blomen_KBM7 > -0.15807728469371796 and

round_StdDev_Transcript_length <= 1275.7037353515625),

-0.010270465248197970312 * (round_Phi <= 3.313508932478726e-05 and

round_missense_Z <= 4.052361965179443),

-0.014236579328625676225 * (round_s_het <= 0.025150161236524582 and

round_Transcript_count > 13.5 and

round_Average_Transcript_length > 1873.0999755859375),

0.081211064552060438504 * (round_End > 85205200.0 and

round_Gene_Length_bp > 9930.5 and

round_StdDev_Transcript_length > 2430.197265625),

0.0089967961046419821919 * (round_Degree <= 12.5 and

round_Degree_mi <= 0.5 and

round_missense_Z <= 2.120723009109497 and

round_StdDev_Transcript_length > 1011.6593627929688),

-0.12108937018609394753 * (not Group == u'MNC' and

round_Tajima__s_D_regulatory <= -0.4115000069141388 and

round_Gene_Length_bp <= 8050.5),

0.081999324716289165305 * (round_Closeness > 0.32499998807907104 and

round_Tajima__s_D_regulatory <= -1.2874999046325684 and

round_Blomen_KBM7 <= -0.41121259331703186),

-0.04692241815911364633 * (round_Closeness <= 0.3149999976158142 and

round_s_het <= 0.025150161236524582 and

round_Average_Transcript_length > 2057.064453125),

0.043127172504397952302 * (round_End > 100419720.0 and

round_Tajima__s_D_regulatory > 0.5145000219345093 and

round_missense_Z <= 3.996763229370117 and

round_StdDev_Transcript_length > 166.57864379882812),

-0.02368886292594349699 * (not Group == u'CM' and

round_End > 100419720.0 and

round_Degree <= 12.5 and

round_Blomen_KBM7 > -0.514844536781311),

0.012990241102245232707 * (round_Closeness > 0.2549999952316284 and

round_Phi > 0.4316735863685608),

-0.0046364819849940520566 * (round_Tajima__s_D_regulatory_mi),

0.0051529804551414338035 * (round_Degree > 3.5 and

round_dN_dS_Chimp <= 0.5950000286102295 and

round_Tajima__s_D_regulatory <= 0.18650001287460327 and

round_Gene_Length_bp <= 343581.5),

0.0438080419095184595 * (not Group == u'NDNE' and

round_Degree > 12.5 and

round_Closeness > 0.32499998807907104 and

round_Gene_Length_bp > 53079.0),

-0.024672830644793142252 * (not Group == u'NDNE' and

round_s_het > 0.016313210129737854 and

round_Gene_Length_bp <= 38001.5),

0.02030768844142512991 * (round_missense_Z <= 4.052361965179443 and

round_LofTool <= 0.04450000077486038 and

round_s_het <= 0.014458265155553818),

0.021488441370476205755 * (not Group == u'MNC' and

round_Closeness > 0.3149999976158142 and

round_StdDev_Transcript_length > 899.939453125),

-0.0377281103161658804 * (Group == u'NDNE' and

round_Degree > 3.5 and

round_Phi <= 0.919446587562561),

-0.047992281795421115609 * (round_Gene_Length_bp <= 2599.0 and

round_StdDev_Transcript_length <= 166.57864379882812),

-0.0030180344075642685266 * (round_Closeness <= 0.3149999976158142 and

round_dN_dS_Chimp <= 0.5950000286102295 and

round_Tajima__s_D_regulatory > 0.12399999797344208 and

round_StdDev_Transcript_length > 1944.22802734375),

-0.053645815472417320013 * (round_StdDev_Transcript_length <= 169.24288940429688 and

round_Average_Transcript_length <= 2318.75),

0.005484710865094958622 * (round_Degree > 3.5 and

round_Closeness > 0.3149999976158142 and

round_s_het > 0.02518850564956665 and

round_Transcript_count <= 15.5),

0.01851729216318677082 * (not Group == u'NDNE' and

round_Degree <= 4.5),

-0.009235200960148456234 * (Group == u'MNC' and

round_Tajima__s_D_regulatory > -1.2874999046325684),

0.0048887605175608542241 * (not Group == u'NDNE' and

round_Degree > 3.5 and

round_Closeness <= 0.3149999976158142 and

round_Phi > 0.8785387277603149),

0.017492794284271317301 * (round_missense_Z <= 4.122845649719238 and

2814.5 < round_Gene_Length_bp <= 9338.5 and

round_StdDev_Transcript_length <= 580.992431640625),

0.0021128405691882255757 * (Group == u'NDNE' and

round_s_het > 0.017306189984083176 and

round_StdDev_Transcript_length > 621.5349731445312 and

round_Exon_Count > 171.0),

-0.028339346103928231974 * (not Group == u'NDNE' and

round_End <= 100419720.0 and

round_Phi > 0.998741626739502 and

round_Transcript_count <= 8.5),

2.8544422492723128641E-05 * (round_missense_Z <= 3.2976694107055664 and

round_Transcript_count > 25.5 and

round_Gene_Length_bp > 9941.5 and

round_Average_Transcript_length <= 2071.857421875),

-0.0072243283196216957764 * (round_Degree <= 12.5 and

round_dN_dS_Chimp_mi <= 0.5 and

round_LofTool > 0.6634999513626099),

-0.022293006054387030229 * (round_LofTool <= 0.6634999513626099 and

round_s_het > 0.01709270477294922 and

round_Exon_Count <= 88.5),

0.022977805449827696377 * (not Group == u'NDNE' and

round_Closeness > 0.2549999952316284 and

round_Transcript_count <= 15.5),

-0.017991002487537172821 * (not Group == u'CM' and

round_Tajima__s_D_regulatory > 0.17550000548362732 and

round_Transcript_count <= 10.5 and

round_Gene_Length_bp > 2814.5),

0.13358397646296524264 * (round_Degree_mi <= 0.5 and

round_LofTool > 0.9907699823379517),

-0.049028952313931860318 * (round_Phi <= 0.12447576969861984 and

83.5 < round_Exon_Count <= 242.5),

0.0021989595530416358206 * (round_missense_Z),

0.021122374812430694951 * (round_Blomen_KBM7 <= -0.157728910446167 and

round_missense_Z > 3.2971019744873047 and

round_Exon_Count > 96.5),

0.040975473907031947918 * (round_LofTool > 0.9921150207519531 and

round_s_het > 0.025150161236524582),

-0.029039626470798590024 * (not Group == u'NDNE' and

round_Closeness <= 0.2549999952316284 and

round_Phi <= 0.8768634796142578),

-0.0093112986032186061125 * (round_Gene_Length_bp > 9978.0 and

round_StdDev_Transcript_length <= 2377.90625 and

round_Exon_Count <= 87.5),

-0.010142192141983820061 * (round_End <= 100419720.0 and

round_Closeness <= 0.3149999976158142 and

round_dN_dS_Chimp <= 0.5950000286102295 and

round_Exon_Count <= 221.5),

-0.073112609476678830367 * (round_Blomen_KBM7_mi),

-0.0097552048672122273348 * (round_Phi <= 0.1378774642944336 and

round_LofTool <= 0.659500002861023 and

round_Average_Transcript_length <= 2515.3544921875),

0.02224374309944953873 * (round_dN_dS_Chimp <= 0.5950000286102295 and

3.2976694107055664 < round_missense_Z <= 4.052361965179443 and

round_LofTool <= 0.9922449588775635),

-0.023271586599581565308 * (round_s_het),

-0.01302008707847174436 * (round_Degree_mi <= 0.5 and

round_missense_Z > 2.552614450454712 and

round_Average_Transcript_length <= 2570.535888671875),

-0.0089483655310949161005 * (round_End <= 100419720.0 and

round_missense_Z > 4.031624794006348 and

round_StdDev_Transcript_length > 166.57864379882812 and

round_Exon_Count <= 221.5),

-0.0076119255564191818514 * (9338.5 < round_Gene_Length_bp <= 37833.5 and

round_StdDev_Transcript_length <= 1956.23095703125),

-0.1477711271932482251 * (round_Degree_mi > 0.5 and

round_Tajima__s_D_regulatory <= 0.47749999165534973),

-0.15850568711767087926 * (not Group == u'MNC' and

round_Tajima__s_D_regulatory > -0.4115000069141388 and

9988.0 < round_Gene_Length_bp <= 53048.5),

-0.027338158540968542087 * (not Group == u'NDNE' and

round_Degree <= 12.5 and

round_missense_Z <= 3.2976694107055664 and

round_LofTool <= 0.9905250072479248),

0.10791704802889216797 * (round_Degree > 35.5 and

round_dN_dS_Chimp <= 0.5950000286102295 and

round_Blomen_KBM7 <= -0.12487166374921799 and

round_missense_Z <= 4.052361965179443),

0.038052681275016403406 * (round_Degree_mi <= 0.5 and

round_Blomen_KBM7 <= -0.15531033277511597 and

round_missense_Z > 2.721888303756714 and

round_Exon_Count <= 262.0),

0.032420599714973505345 * (round_Degree > 12.5 and

round_s_het > 0.02518850564956665 and

round_Exon_Count <= 284.0),

0.068969116586947529224 * (round_Degree > 35.5 and

round_Degree_mi <= 0.5 and

round_Blomen_KBM7 > -0.1796257197856903 and

round_LofTool <= 0.874500036239624),

0.011001837801926907245 * (round_s_het > 0.02518850564956665 and

round_Transcript_count > 15.5),

0.032911944288080231813 * (not Group == u'NDNE' and

round_Degree > 3.5 and

round_Phi > 0.4623493552207947 and

round_LofTool <= 0.9787000417709351),

0.056478680464096327196 * (not Group == u'NDNE' and

round_Degree <= 12.5 and

round_s_het > 0.02518850564956665 and

round_Exon_Count > 160.5),

-0.033531490400225016923 * (not Group == u'NDNE' and

round_Degree > 4.5 and

round_Degree_mi <= 0.5 and

round_Phi <= 0.919446587562561),

0.032944099199805537692 * (-1.2874999046325684 < round_Tajima__s_D_regulatory <= 1.4165000915527344 and

round_missense_Z <= 4.028769493103027 and

round_Transcript_count > 20.5),

0.028319937430381268706 * (round_Degree > 12.5 and

round_Closeness <= 0.33500000834465027 and

round_Tajima__s_D_regulatory <= 0.4235000014305115 and

round_LofTool <= 0.9922800064086914),

-0.0026354135038181500383 * (round_Closeness <= 0.3149999976158142 and

round_Blomen_KBM7 <= -0.24819540977478027 and

round_missense_Z <= 3.2976694107055664 and

round_StdDev_Transcript_length > 169.24288940429688),

-0.0070229179471281545991 * (round_Degree_mi <= 0.5 and

round_Closeness <= 0.3149999976158142 and

round_Tajima__s_D_regulatory <= 0.4235000014305115 and

round_Exon_Count <= 156.5),

-0.42263729054912357874 * (Group == u'NDNE' and

round_Degree <= 3.5 and

round_StdDev_Transcript_length > 714.263427734375),

0.083714906610359116068 * (round_Closeness > 0.3149999976158142 and

round_Transcript_count > 19.5),

-0.024520343423184185611 * (round_missense_Z <= 0.4934942126274109 and

round_Exon_Count <= 96.5),

0.010109225596981909548 * (not Group == u'NDNE' and

round_Closeness > 0.3149999976158142 and

round_Phi > 0.12447576969861984 and

round_Exon_Count <= 218.5),

0.0032675836626067386939 * (round_Degree_mi <= 0.5 and

round_missense_Z <= 3.335038185119629 and

round_StdDev_Transcript_length > 2377.02734375),

0.00060752664301474447461 * (Group == u'NDNE' and

round_Degree <= 4.5 and

round_StdDev_Transcript_length > 714.263427734375),

-0.010258215185811834017 * (round_Degree_mi <= 0.5 and

round_Closeness <= 0.2549999952316284 and

round_Phi <= 0.4316735863685608 and

round_Transcript_count <= 9.5),

0.03375802518351931486 * (not Group == u'MNC' and

round_Closeness > 0.33500000834465027 and

3.2976694107055664 < round_missense_Z <= 4.050085067749023),

-0.012795976026728770811 * (round_Degree <= 12.5 and

round_Closeness <= 0.7100000381469727 and

round_missense_Z <= 4.0613203048706055 and

round_Gene_Length_bp <= 352096.0),

0.0023318898906593155812 * (round_Closeness > 0.2549999952316284 and

round_Phi <= 0.4316735863685608 and

round_Transcript_count <= 9.5),

-0.0066755401432419267729 * (round_Degree <= 12.5 and

round_Closeness <= 0.3050000071525574 and

round_dN_dS_Chimp_mi <= 0.5 and

round_StdDev_Transcript_length > 168.6014404296875),

-0.0034145756247275114645 * (round_LofTool <= 0.6634999513626099 and

round_s_het <= 0.01709270477294922 and

round_Exon_Count <= 88.5),

0.020834681193880762867 * (round_Tajima__s_D_regulatory <= 1.4165000915527344 and

round_Blomen_KBM7 <= -0.5153244733810425 and

round_Transcript_count > 10.5 and

round_Gene_Length_bp > 10640.5),

-0.001953615307148606154 * (round_Phi <= 0.12447576969861984 and

round_LofTool > 0.9605500102043152 and

round_StdDev_Transcript_length > 1452.739013671875),

0.014062538630450481178 * (Group == u'NDNE' and

round_Degree_mi <= 0.5 and

round_Average_Transcript_length > 2570.535888671875),

0.0052869251864379315439 * (round_Phi > 0.00015248148702085018 and

round_missense_Z <= 3.2976694107055664 and

round_Exon_Count <= 87.5),

-0.02333450869429674196 * (not Group == u'MNC' and

round_Tajima__s_D_regulatory > 1.2934999465942383 and

round_Blomen_KBM7 > -0.4999815821647644),

-0.0013849296134431871106 * (Group == u'NDNE' and

round_s_het > 0.01700003445148468 and

round_StdDev_Transcript_length <= 704.5946044921875 and

round_Exon_Count <= 86.5),

0.011101717126482805661 * (round_Degree <= 12.5 and

round_s_het > 0.017613736912608147 and

round_Gene_Length_bp > 38001.5 and

round_Exon_Count > 221.5),

0.070135120685213117597 * (round_Closeness > 0.3149999976158142 and

round_Phi <= 0.8754478693008423),

-0.064495788823159316827 * (not Group == u'NDNE' and

round_Closeness <= 0.2549999952316284 and

round_LofTool <= 0.8554999828338623),

0.00050612016669284083138 * (round_End <= 100419720.0 and

round_Tajima__s_D_regulatory > -1.2885000705718994 and

round_missense_Z_mi > 0.5),

-0.073034808226031561196 * (round_Gene_Length_bp > 2599.0 and

round_StdDev_Transcript_length <= 166.57864379882812),

-0.032090827359876047953 * (Group == u'NDNE' and

round_missense_Z > 2.7258143424987793),

0.040659733289495375574 * (round_Tajima__s_D_regulatory <= -1.2874999046325684 and

round_missense_Z <= 3.992063522338867 and

round_Exon_Count <= 224.5),

0.026721043939407669587 * (not Group == u'NDNE' and

round_Closeness > 0.2549999952316284 and

round_LofTool <= 0.9787000417709351 and

round_StdDev_Transcript_length <= 1005.2410888671875),

0.0091395249084863204592 * (not Group == u'MNC' and

round_Degree > 5.5 and

round_Phi > 0.0015477617271244526 and

round_Blomen_KBM7 <= -0.18366125226020813),

0.0060628959549816306696 * (round_End <= 127637464.0 and

round_dN_dS_Chimp_mi <= 0.5 and

round_Gene_Length_bp > 9941.5 and

round_Average_Transcript_length <= 2037.5650634765625),

-0.039510142557001665109 * (round_Tajima__s_D_regulatory > -1.2874999046325684 and

round_missense_Z > 3.8540451526641846),

0.019811125846081169277 * (round_Degree <= 4.5 and

round_missense_Z <= 4.0604472160339355 and

round_Transcript_count <= 20.5 and

round_StdDev_Transcript_length > 1967.8238525390625),

-0.0018599456342518888574 * (Group == u'NDNE' and

round_End <= 100419720.0 and

round_Tajima__s_D_regulatory > -1.2885000705718994 and

round_Blomen_KBM7 <= -0.09869569540023804),

0.0021562156312482580467 * (not Group == u'MNC' and

round_dN_dS_Chimp_mi <= 0.5 and

round_Blomen_KBM7 > -0.5146373510360718 and

round_Gene_Length_bp > 37999.5),

-0.049805293559377718238 * (Group == u'NDNE' and

round_StdDev_Transcript_length <= 621.5349731445312),

0.014492269940647846405 * (round_End > 100419720.0 and

round_missense_Z > 4.076244354248047 and

round_LofTool <= 0.994350016117096),

0.1236092272944794429 * (round_LofTool > 0.9922449588775635),

-0.031030338902052499728 * (Group == u'NDNE' and

round_Degree <= 12.5 and

round_Average_Transcript_length <= 2169.2587890625),

0.026392057958906565279 * (round_Blomen_KBM7 > -0.49977701902389526 and

round_LofTool > 0.9922449588775635),

0.065464842259792752066 * (round_LofTool <= 0.9920099973678589 and

round_Exon_Count > 221.5),

0.0055693260621947279776 * (round_Degree > 3.5 and

round_Closeness <= 0.3149999976158142 and

round_Exon_Count > 151.5),

-0.012235121495508578457 * (not Group == u'NDNE' and

round_Closeness <= 0.2549999952316284 and

round_s_het > 0.021862218156456947),

0.019996450446880205398 * (round_Tajima__s_D_regulatory > 0.4235000014305115 and

round_Blomen_KBM7 <= -0.21081802248954773 and

round_s_het > 0.015080630779266357 and

round_StdDev_Transcript_length > 580.992431640625),

-0.0010861882066284769995 * (Group == u'NDNE' and

round_Degree > 4.5 and

round_Degree_mi <= 0.5 and

round_Phi > 0.919446587562561),

-0.020932057542549405149 * (round_dN_dS_Chimp_mi > 0.5 and

round_Tajima__s_D_regulatory > -1.2909998893737793),

0.0043345273324120409467 * (not Group == u'MNC' and

round_dN_dS_Chimp_mi > 0.5 and

round_Tajima__s_D_regulatory > -1.2874999046325684 and

round_Blomen_KBM7 <= -0.4999815821647644),

-0.0056254294468995819437 * (not Group == u'CM' and

not Group == u'MNC' and

-1.2855000495910645 < round_Tajima__s_D_regulatory <= 1.4165000915527344),

-0.051135930907465425299 * (round_missense_Z <= 3.04426908493042 and

round_StdDev_Transcript_length <= 167.33212280273438),

0.0012261433532188758741 * (round_Degree > 4.5 and

round_Phi > 0.12357446551322937 and

round_Blomen_KBM7 <= -0.15807728469371796 and

round_StdDev_Transcript_length <= 1275.7037353515625),

0.023396898187144792025 * (not Group == u'NDNE' and

round_Closeness <= 0.3149999976158142 and

round_Phi > 0.12447576969861984 and

round_Gene_Length_bp > 45328.0),

-0.00073349258122191681765 * (round_Degree > 4.5 and

round_Closeness <= 0.3149999976158142 and

round_StdDev_Transcript_length > 1275.7037353515625),

0.001877324657792885142 * (round_Degree <= 9.5 and

round_Phi > 0.1378774642944336 and

round_LofTool <= 0.659500002861023),

-7.2347323261013874146E-06 * (round_Exon_Count),

-0.0046239247092063531092 * (round_dN_dS_Chimp_mi <= 0.5 and

round_missense_Z <= 3.2976694107055664 and

round_StdDev_Transcript_length > 166.57864379882812 and

round_Average_Transcript_length > 2071.857421875),

-0.090305482829968683478 * (round_Phi <= 0.0002437000221107155 and

round_missense_Z <= 4.0604472160339355 and

round_StdDev_Transcript_length > 615.416259765625),

0.01648482491782999812 * (round_Degree <= 45.0 and

round_dN_dS_Chimp_mi > 0.5 and

round_Transcript_count <= 26.5 and

round_Gene_Length_bp > 2402.0),

-0.025610478117531781939 * (not Group == u'NDNE' and

round_Degree <= 57.5 and

round_missense_Z <= 2.642360210418701 and

round_Exon_Count <= 190.5),

0.013462495283131365245 * (not Group == u'CM' and

not Group == u'MNC' and

round_Degree > 70.0 and

round_LofTool > 0.8695000410079956),

0.010003967660577692267 * (round_dN_dS_Chimp_mi > 0.5 and

round_s_het <= 0.43051624298095703 and

round_Gene_Length_bp > 2821.0),

-0.25087679754859976144 * (round_Phi > 0.00015248148702085018 and

round_Blomen_KBM7 <= -0.15492522716522217 and

round_LofTool <= 0.6634999513626099 and

round_Exon_Count <= 157.5),

-0.20988131906886342559 * (round_Degree_mi > 0.5 and

round_Tajima__s_D_regulatory > 0.47749999165534973),

0.014989444923831543588 * (Group == u'MNC' and

round_Phi > 0.0015477617271244526 and

round_Exon_Count > 96.5),

-0.0083027455186904398216 * (not Group == u'MNC' and

round_Degree <= 5.5 and

round_Gene_Length_bp <= 43309.0),

-0.16073662255780979402 * (round_LofTool <= 0.6634999513626099 and

round_Exon_Count > 157.5),

0.035010641447032482543 * (2630.5 < round_Gene_Length_bp <= 9978.0 and

round_StdDev_Transcript_length <= 2377.90625),

-0.021649303102464188125 * (round_missense_Z <= 3.3354156017303467 and

round_StdDev_Transcript_length <= 636.7213134765625),

-0.02887978211493740649 * (round_missense_Z > 3.3354156017303467 and

round_StdDev_Transcript_length <= 636.7213134765625),

-0.015589426309587621142 * (not Group == u'CM' and

round_dN_dS_Chimp_mi <= 0.5 and

round_Tajima__s_D_regulatory > 1.062999963760376 and

round_Blomen_KBM7_mi <= 0.5),

0.007869031006701419223 * (not Group == u'NDNE' and

round_Tajima__s_D_regulatory > 0.5564999580383301),

0.01919846955873811753 * (round_Closeness <= 0.2549999952316284 and

round_missense_Z <= 4.052361965179443 and

round_s_het > 0.014458265155553818 and

round_s_het_mi > 0.5),

-0.013523761746092448702 * (round_Closeness > 0.2549999952316284 and

round_missense_Z <= 1.43977952003479 and

round_StdDev_Transcript_length <= 902.3864135742188 and

round_Exon_Count <= 260.5),

0.056557835112290621993 * (not Group == u'NDNE' and

round_Degree > 4.5 and

round_LofTool > 0.9827499985694885),

1.077170414030006006E-06 * (round_StdDev_Transcript_length),

0.0067367214229233718728 * (not Group == u'NDNE' and

round_Closeness > 0.32499998807907104 and

round_missense_Z > 1.43977952003479),

-0.025280204319459625983 * (round_Blomen_KBM7 > -0.24819540977478027 and

round_missense_Z <= 3.2976694107055664),

-0.07144481186709027154 * (Group == u'NDNE' and

round_missense_Z <= 1.1351158618927002 and

round_Transcript_count <= 10.5),

-0.034050179534380775603 * (round_End <= 100419720.0 and

round_Closeness > 0.3149999976158142 and

round_dN_dS_Chimp <= 0.5950000286102295 and

round_missense_Z <= 2.852269172668457),

0.038061134138614192979 * (not Group == u'MNC' and

round_missense_Z <= 2.642360210418701 and

round_s_het > 0.014316117390990257 and

round_Exon_Count > 165.5),

-0.076712140314540558372 * (round_Closeness > 0.2549999952316284 and

round_Phi > 0.0002437000221107155 and

round_missense_Z <= 4.0604472160339355 and

round_StdDev_Transcript_length > 615.416259765625),

-0.039402444954045441616 * (round_Closeness <= 0.3149999976158142 and

round_dN_dS_Chimp > 0.5950000286102295),

-0.084793394898434820695 * (Group == u'NDNE' and

round_Degree > 4.5 and

round_Phi <= 0.13842733204364777),

-0.012276591717802870854 * (round_Closeness <= 0.33500000834465027 and

round_Blomen_KBM7 > -0.49977701902389526 and

round_missense_Z > 4.052361965179443 and

round_LofTool <= 0.9922449588775635),

0.0049006772599034468391 * (round_End <= 127093776.0 and

round_Tajima__s_D_regulatory > -1.2894999980926514 and

round_Blomen_KBM7 <= -0.49977701902389526 and

round_Exon_Count <= 218.5),

-0.017059843137643783406 * (0.659500002861023 < round_LofTool <= 0.9921150207519531 and

round_StdDev_Transcript_length <= 589.7366333007812 and

round_Exon_Count <= 174.5),

-0.0075798691384530445317 * (round_End <= 100419720.0 and

round_dN_dS_Chimp_mi <= 0.5 and

round_Blomen_KBM7_mi <= 0.5 and

round_Exon_Count <= 221.5),

0.010744566051742131946 * (12.5 < round_Degree <= 60.5 and

round_Tajima__s_D_regulatory <= 1.0529999732971191),

-0.38591243129541930035 * (Group == u'NDNE' and

round_Closeness <= 0.2549999952316284 and

round_Phi > 0.14362311363220215),

-0.040250397690003617002 * (not Group == u'NDNE' and

round_Phi <= 0.12447576969861984 and

round_Exon_Count <= 83.5),

0.0054042513292536526609 * (not Group == u'NDNE' and

round_Phi > 0.12447576969861984 and

round_Exon_Count <= 93.5),

0.012640846218055473704 * (round_Degree > 4.5 and

round_Blomen_KBM7 <= -0.1469864696264267 and

round_missense_Z > 4.099565505981445 and

round_Exon_Count <= 224.5),

-0.011599627423029612583 * (not Group == u'CM' and

3.5 < round_Degree <= 56.5 and

round_Exon_Count <= 253.5),

0.024923686490065564969 * (round_dN_dS_Chimp_mi <= 0.5 and

round_Phi <= 0.9998619556427002 and

round_StdDev_Transcript_length > 2625.484375),

-0.024788357238958731721 * (not Group == u'CM' and

not Group == u'MNC' and

round_Tajima__s_D_regulatory <= 0.4104999899864197 and

round_Transcript_count <= 20.5),

-0.032616344153410088691 * (4.5 < round_Degree <= 29.5 and

round_StdDev_Transcript_length <= 897.559326171875),

-0.035027548355530679913 * (Group == u'NDNE' and

round_Degree <= 3.5 and

round_StdDev_Transcript_length > 1049.45654296875),

-0.0074999311629464779708 * (Group == u'NDNE' and

round_dN_dS_Chimp <= 0.5950000286102295 and

round_Transcript_count <= 20.5 and

round_StdDev_Transcript_length <= 1967.8238525390625),

0.033847444063908105338 * (round_Degree > 12.5 and

round_LofTool <= 0.9921150207519531 and

round_s_het > 0.025150161236524582 and

round_Exon_Count <= 224.5),

0.018672957826625806443 * (round_Phi <= 0.00015248148702085018 and

round_missense_Z <= 4.052361965179443 and

round_LofTool > 0.11749999970197678),

-0.0066963862081202731036 * (round_Degree_mi <= 0.5 and

round_Closeness <= 0.3149999976158142 and

round_Tajima__s_D_regulatory > 0.4235000014305115 and

round_Exon_Count <= 156.5),

-0.19403437417903002249 * (round_Degree <= 4.5 and

round_Phi > 0.14253592491149902),

0.025474159388727657394 * (round_Degree > 4.5 and

round_Closeness > 0.3149999976158142 and

round_StdDev_Transcript_length > 1275.7037353515625),

-0.15200914772366930228 * (round_Gene_Length_bp <= 2814.5),

-0.012369173690002560964 * (Group == u'NDNE' and

round_Degree_mi <= 0.5 and

round_missense_Z <= 2.7698380947113037 and

round_Gene_Length_bp <= 39341.0),

0.019096003975550827902 * (round_Degree <= 4.5 and

round_Degree_mi <= 0.5 and

round_StdDev_Transcript_length <= 1301.4395751953125),

-0.060655818768655632434 * (round_Blomen_KBM7 > -0.5146373510360718 and

round_Transcript_count <= 20.5 and

round_StdDev_Transcript_length > 2388.5126953125),

-0.022501726195653822676 * (not Group == u'NDNE' and

round_Degree <= 62.5 and

round_Transcript_count <= 20.5 and

round_StdDev_Transcript_length <= 580.6193237304688),

0.06316842250916585022 * (not Group == u'NDNE' and

round_Degree > 3.5 and

round_Phi <= 0.8768634796142578 and

round_LofTool <= 0.9829000234603882),

0.039685738909723246304 * (Group == u'NDNE' and

round_Degree <= 3.5 and

round_missense_Z > 1.472226858139038),

-0.002509813077558311397 * (round_Degree_mi <= 0.5 and

round_Closeness <= 0.32499998807907104 and

round_LofTool <= 0.9435499906539917 and

round_Gene_Length_bp <= 54517.0),

-0.039332187759655926063 * (round_dN_dS_Chimp > 0.5950000286102295),

-0.06643661699653181929 * (Group == u'NDNE' and

round_Closeness > 0.2549999952316284),

-0.021412501080032213946 * (round_Degree > 3.5 and

round_Phi <= 0.12447576969861984 and

round_Exon_Count <= 156.5),

-0.00029745582835470673125 * (round_Transcript_count),

0.057181720796839995147 * (round_Closeness > 0.3149999976158142 and

round_Phi > 0.12447576969861984),

-1.1772852955159365113E-05 * (round_Tajima__s_D_regulatory <= 0.4165000021457672 and

round_Phi > 0.00015248148702085018 and

round_missense_Z <= 3.308867931365967),

-0.078557558657171636107 * (round_dN_dS_Chimp_mi <= 0.5 and

round_missense_Z <= 4.0604472160339355 and

round_StdDev_Transcript_length <= 615.416259765625),

0.017463172080480923037 * (round_Closeness > 0.2549999952316284 and

round_LofTool > 0.9937300086021423 and

round_Gene_Length_bp > 39268.5),

-0.00041244386195987383292 * (round_Degree <= 60.5 and

round_dN_dS_Chimp_mi > 0.5 and

round_Blomen_KBM7 > -0.5153281092643738 and

round_Exon_Count <= 154.5),

-0.012984409356028072183 * (round_Degree > 4.5 and

round_Phi > 0.0003650499857030809 and

round_Blomen_KBM7 <= -0.1469864696264267 and

round_missense_Z <= 4.099565505981445),

-0.013769490895467194 * (not Group == u'MNC' and

round_Degree <= 34.5 and

round_Degree_mi <= 0.5 and

round_missense_Z <= 4.0604472160339355),

0.026191082501492171652 * (round_missense_Z <= 2.695338487625122 and

round_Transcript_count > 26.5 and

round_StdDev_Transcript_length > 169.24288940429688),

-0.0087539696043418078336 * (not Group == u'CM' and

round_Closeness <= 0.7100000381469727 and

round_Blomen_KBM7 <= -0.49977701902389526 and

round_LofTool <= 0.6634999513626099),

0.015364239969804956848 * (round_Degree > 37.5 and

round_Degree_mi <= 0.5 and

round_Blomen_KBM7 <= -0.24797455966472626 and

round_missense_Z <= 2.721888303756714),

-0.013768597282704474888 * (round_Degree > 5.5 and

round_Blomen_KBM7 <= -0.15774735808372498 and

round_Average_Transcript_length <= 2566.631103515625),

0.0016667767258665866937 * (Group == u'NDNE' and

round_Phi > 0.12447576969861984 and

round_Exon_Count <= 93.5),

0.18128583819906532448 * (round_Closeness > 0.3149999976158142 and

round_Blomen_KBM7 <= -0.5528146028518677 and

round_Exon_Count <= 224.5),

0.012943950146806795093 * (round_Degree > 12.5 and

round_dN_dS_Chimp_mi <= 0.5 and

round_Blomen_KBM7 <= -0.2521226406097412 and

round_Gene_Length_bp > 2814.5),

-0.0049955332334434728037 * (round_LofTool),

0.014164001392438506019 * (round_Blomen_KBM7 <= -0.5153281092643738 and

round_missense_Z > 3.289116144180298),

0.050500102827979391484 * (round_End > 124883040.0 and

-1.2874999046325684 < round_Tajima__s_D_regulatory <= 1.4165000915527344 and

round_Transcript_count <= 20.5),

0.060456640460729016429 * (not Group == u'NDNE' and

round_Degree <= 12.5 and

round_StdDev_Transcript_length > 636.7213134765625 and

round_Exon_Count <= 253.5),

-0.033822224075698618939 * (round_Blomen_KBM7),

-0.0039964088486853953028 * (round_Closeness <= 0.33500000834465027 and

round_Blomen_KBM7 > -0.49977701902389526 and

round_missense_Z <= 4.052361965179443 and

round_LofTool <= 0.9922449588775635),

0.023975111109943367249 * (round_End <= 85205200.0 and

round_Gene_Length_bp > 9930.5 and

round_StdDev_Transcript_length > 2430.197265625),

-0.011463148479432971882 * (round_Tajima__s_D_regulatory > 1.0544999837875366 and

round_missense_Z <= 4.052361965179443 and

round_Average_Transcript_length <= 2288.774658203125),

-0.0051712503030842075363 * (round_Degree_mi <= 0.5 and

round_missense_Z <= 2.552614450454712 and

round_LofTool <= 0.6644999980926514 and

round_Average_Transcript_length <= 2570.535888671875),

0.037739634085657752793 * (round_missense_Z > 3.2976694107055664),

-0.021570209135655677574 * (round_Closeness > 0.3149999976158142 and

round_Phi <= 0.12447576969861984 and

round_Exon_Count <= 83.5),

-0.021510107011690166728 * (not Group == u'NDNE' and

round_Closeness <= 0.2549999952316284 and

round_Phi > 0.9640257358551025),

0.0014163650512331741119 * (round_Degree <= 12.5 and

round_missense_Z > 3.2976694107055664),

-0.010674563779895991297 * (4.050085067749023 < round_missense_Z <= 5.5646071434021),

0.041860350492729625493 * (round_Degree > 35.5 and

round_Degree_mi <= 0.5 and

round_Closeness > 0.2549999952316284 and

round_Phi <= 0.8162848949432373),

0.0056832531972258086908 * (round_dN_dS_Chimp_mi <= 0.5 and

round_Transcript_count > 26.5 and

round_StdDev_Transcript_length > 169.24288940429688),

-0.063871267111750978929 * (round_Degree_mi > 0.5 and

round_Phi <= 0.8162848949432373),

0.034891184078606619912 * (3.5 < round_Degree <= 63.0 and

round_Phi > 0.919446587562561),

-0.0018919554407697701439 * (round_Phi <= 0.0015477617271244526 and

round_Gene_Length_bp > 50399.0),

0.013992933384094302304 * (round_Phi > 0.0023738450836390257 and

round_Blomen_KBM7 <= -0.18453440070152283 and

round_Transcript_count > 26.5 and

round_Exon_Count > 87.5),

-0.051195693008280626635 * (round_Degree <= 3.5 and

round_s_het <= 0.02518850564956665),

0.055834058359271090954 * (not Group == u'NDNE' and

round_Degree <= 12.5 and

round_s_het > 0.02518850564956665 and

round_Exon_Count <= 160.5),

-0.0082870956775982305281 * (round_Closeness <= 0.2549999952316284 and

round_Tajima__s_D_regulatory > 0.4104999899864197),

0.038547688444224244286 * (Group == u'MNC' and

round_Closeness > 0.3149999976158142),

0.013900893462213393531 * (round_Closeness > 0.2549999952316284 and

round_LofTool > 0.7979999780654907 and

round_Gene_Length_bp <= 41216.0),

0.0050657262704566644387 * (round_Closeness > 0.2549999952316284 and

round_Phi <= 0.00013825652422383428 and

round_missense_Z <= 3.334120750427246),

-0.018169172998649386203 * (round_End <= 100419720.0 and

round_Degree_mi > 0.5),

0.0044089773122458876878 * (round_Tajima__s_D_regulatory > -1.2874999046325684 and

round_Blomen_KBM7 > -0.12487166374921799 and

round_s_het > 0.02633928880095482 and

round_Exon_Count > 53.0),

-0.071852306419135800186 * (round_dN_dS_Chimp_mi > 0.5 and

round_missense_Z <= 4.0604472160339355 and

round_StdDev_Transcript_length <= 615.416259765625),

0.073788341751617983477 * (round_Degree > 60.5 and

round_Tajima__s_D_regulatory <= 1.0529999732971191),

-0.0019138125607677928565 * (round_Blomen_KBM7 > -0.5153281092643738 and

round_missense_Z <= 4.103667736053467 and

round_Transcript_count <= 24.5 and

round_Gene_Length_bp > 53047.5),

0.0038228233805468426684 * (round_Closeness <= 0.3149999976158142 and

round_missense_Z <= 2.571293354034424 and

round_StdDev_Transcript_length > 1450.0491943359375),

0.0097863017065103925785 * (not Group == u'NDNE' and

round_Closeness <= 0.3149999976158142 and

round_Phi > 0.12447576969861984 and

round_Exon_Count <= 218.5),

-0.064557916522001199122 * (round_Degree_mi > 0.5 and

round_Gene_Length_bp > 2510.5),

0.017207469487847969897 * (not Group == u'NDNE' and

round_Degree_mi <= 0.5 and

round_LofTool <= 0.9829000234603882 and

round_Average_Transcript_length > 1918.333251953125),

0.016078813237064535496 * (round_Tajima__s_D_regulatory <= 0.195499986410141 and

round_missense_Z > 3.2976694107055664),

0.0054438014970486035132 * (round_Closeness <= 0.2549999952316284 and

round_missense_Z <= 4.052361965179443 and

round_s_het > 0.014458265155553818 and

round_s_het_mi <= 0.5),

-0.056622008362259834691 * (round_Degree <= 3.5 and

0.6634999513626099 < round_LofTool <= 0.9905250072479248),

-0.009382110839505006239 * (round_Closeness <= 0.26499998569488525 and

round_LofTool <= 0.8105000257492065 and

round_Gene_Length_bp > 37966.5),

-0.022079473690573211964 * (round_missense_Z <= 3.2976694107055664 and

round_Transcript_count <= 25.5 and

round_Gene_Length_bp > 9941.5 and

round_Average_Transcript_length <= 2071.857421875),

0.026567551484747074092 * (round_Degree_mi > 0.5 and

round_LofTool > 0.6614999771118164),

0.012171923038907185924 * (not Group == u'MNC' and

round_Closeness <= 0.33500000834465027 and

3.2976694107055664 < round_missense_Z <= 4.050085067749023),

-0.0076355905706610317091 * (round_Degree <= 5.5 and

round_LofTool > 0.6554999947547913 and

round_StdDev_Transcript_length <= 1337.2225341796875),

0.16325429384657416665 * (round_Tajima__s_D_regulatory <= 0.4235000014305115 and

round_Blomen_KBM7 <= -0.2521226406097412 and

round_s_het > 0.015603477135300636 and

round_Transcript_count > 21.5),

0.0072661225638375511598 * (round_End <= 100419720.0 and

round_StdDev_Transcript_length > 166.57864379882812 and

round_Exon_Count > 221.5),

0.036323776432227984634 * (round_Degree <= 37.5 and

round_Degree_mi <= 0.5 and

round_Blomen_KBM7 <= -0.24797455966472626 and

round_missense_Z <= 2.721888303756714),

-0.013138779464712927597 * (not Group == u'NDNE' and

round_Degree > 4.5 and

round_LofTool <= 0.656499981880188),

0.019063593314076931334 * (not Group == u'NDNE' and

round_Blomen_KBM7 > -0.5146430730819702 and

round_Transcript_count <= 20.5 and

round_StdDev_Transcript_length <= 2418.21435546875),

-0.022312166919272659327 * (not Group == u'NDNE' and

round_missense_Z > 2.642360210418701 and

round_Exon_Count <= 285.0),

0.046627845220519749392 * (round_Degree_mi > 0.5 and

round_Tajima__s_D_regulatory > 0.47749999165534973 and

round_Blomen_KBM7 <= -0.12487166374921799),

-0.057144599564295774086 * (round_Phi_mi),

-0.042548091410918198463 * (Group == u'MNC' and

167.33212280273438 < round_StdDev_Transcript_length <= 615.8639526367188),

-0.078599340027315722779 * (Group == u'NDNE' and

round_StdDev_Transcript_length <= 714.5440063476562),

-0.035888903100163335735 * (Group == u'NDNE' and

round_Phi <= 0.12447576969861984 and

round_Exon_Count <= 83.5),

0.014099712983019761434 * (round_Tajima__s_D_regulatory > 0.4104999899864197 and

round_Exon_Count > 88.5),

-0.025909213199834423696 * (round_Degree > 4.5 and

round_Closeness <= 0.3149999976158142 and

round_Transcript_count <= 24.5 and

round_StdDev_Transcript_length > 897.559326171875),

-0.0074845359849700426533 * (round_Tajima__s_D_regulatory > 0.43549999594688416 and

round_missense_Z <= 3.2976694107055664 and

round_LofTool <= 0.9603500366210938 and

round_Exon_Count <= 164.5),

-0.01077622969677029946 * (round_Degree_mi > 0.5 and

round_dN_dS_Chimp > 0.23499999940395355 and

round_Tajima__s_D_regulatory <= 0.47749999165534973 and

round_Blomen_KBM7 <= -0.12487166374921799),

-0.30401281812512331859 * (round_Degree <= 4.5 and

round_Phi <= 0.14253592491149902 and

round_StdDev_Transcript_length <= 616.5709228515625),

-0.028990481621997066936 * (round_Phi <= 0.12447576969861984 and

round_Transcript_count > 19.5 and

round_StdDev_Transcript_length > 1098.5848388671875),

-0.18916948110773898484 * (3.134337902069092 < round_missense_Z <= 3.308867931365967 and

round_s_het > 0.015855055302381516),

0.0079327801354993239535 * (round_Blomen_KBM7 <= -0.5154723525047302 and

round_missense_Z <= 2.6123709678649902 and

round_StdDev_Transcript_length > 166.57864379882812),

0.0096322052481131285873 * (not Group == u'NDNE' and

round_Closeness > 0.2549999952316284 and

round_Phi > 0.8162848949432373 and

round_LofTool <= 0.9833999872207642),

-0.010776147984111764111 * (round_dN_dS_Chimp_mi <= 0.5 and

round_missense_Z <= 3.308867931365967 and

round_Average_Transcript_length > 2037.5650634765625),

0.043118916775937328467 * (round_Phi <= 3.313508932478726e-05 and

round_missense_Z <= 4.052361965179443 and

round_LofTool > 0.11749999970197678),

-0.033825661237730453301 * (round_Closeness <= 0.2549999952316284 and

round_s_het <= 0.021862218156456947),

-0.047543359055394369961 * (not Group == u'MNC' and

round_s_het <= 0.025150161236524582 and

round_Average_Transcript_length <= 1873.0999755859375),

0.043005070335481156152 * (round_dN_dS_Chimp_mi <= 0.5 and

round_LofTool > 0.9922449588775635),

-0.0193305402296465971 * (round_Phi > 0.9985461235046387 and

round_Transcript_count <= 10.5 and

round_Gene_Length_bp > 53048.5),

-0.0029334074033163580301 * (round_End <= 127204736.0 and

round_dN_dS_Chimp_mi <= 0.5 and

round_Gene_Length_bp > 2814.5 and

round_Average_Transcript_length <= 2037.5650634765625),

-0.014088246133160155227 * (not Group == u'MNC' and

round_End <= 100419720.0 and

round_Degree <= 12.5 and

round_Blomen_KBM7 <= -0.11068554222583771),

-0.031492051646561769473 * (not Group == u'NDNE' and

round_End <= 100419720.0 and

round_Phi > 0.998741626739502 and

round_Transcript_count > 8.5),

0.064514690954963371805 * (round_End > 100419720.0 and

round_LofTool > 0.994350016117096),

0.0037794234205987689915 * (round_Gene_Length_bp <= 9930.5 and

round_StdDev_Transcript_length > 166.57864379882812),

-0.046813844593473395717 * (Group == u'NDNE' and

round_Transcript_count > 10.5),

0.0047308280825400263192 * (round_Closeness > 0.2549999952316284 and

0.6694999933242798 < round_LofTool <= 0.9937300086021423 and

round_Gene_Length_bp > 39268.5),

0.031799043486228008304 * (round_Phi > 0.00015248148702085018 and

round_Blomen_KBM7 <= -0.5508977174758911 and

round_missense_Z <= 4.0604472160339355 and

round_Exon_Count > 87.5),

0.024599510773753802129 * (Group == u'NDNE' and

round_Degree <= 4.5 and

round_StdDev_Transcript_length > 621.470947265625),

-0.024818309340896429344 * (round_Closeness <= 0.2549999952316284 and

round_LofTool <= 0.8535000085830688 and

round_StdDev_Transcript_length > 617.4835815429688),

-0.022986810189580389463 * (not Group == u'CM' and

round_Degree <= 35.5 and

round_Closeness > 0.3050000071525574 and

round_Blomen_KBM7 > -0.49977701902389526),

0.024818347921128428024 * (round_Closeness <= 0.33500000834465027 and

round_dN_dS_Chimp_mi > 0.5 and

round_Blomen_KBM7 <= -0.2521226406097412 and

round_Gene_Length_bp > 2814.5),

0.030785675449449489971 * (round_Degree > 9.5 and

round_Phi > 0.1378774642944336 and

round_LofTool <= 0.659500002861023),

0.071141168378547961493 * (round_Phi > 0.12447576969861984 and

round_Exon_Count > 218.5),

-0.0033207787269117858969 * (round_Blomen_KBM7 <= -0.14714285731315613 and

round_missense_Z <= 3.2974047660827637 and

round_LofTool <= 0.9922449588775635 and

round_StdDev_Transcript_length > 615.8639526367188),

-0.021924118897895659985 * (not Group == u'CM' and

not Group == u'MNC' and

round_Degree <= 35.5 and

round_Blomen_KBM7 > -0.49977701902389526),

-0.015856059453570228723 * (round_dN_dS_Chimp > 0.5950000286102295 and

round_LofTool > 0.04450000077486038 and

round_Exon_Count <= 217.0),

0.055378231519672874161 * (round_LofTool_mi),

-0.0061308536874806300598 * (0.3050000071525574 < round_Closeness <= 0.3149999976158142 and

round_Blomen_KBM7 > -0.5158457159996033 and

round_Gene_Length_bp <= 53140.5),

-0.021087248754085095859 * (not Group == u'CM' and

round_Tajima__s_D_regulatory > 0.4165000021457672 and

round_missense_Z <= 4.031401634216309 and

round_LofTool > 0.6634999513626099),

-0.028317726879795258876 * (round_Degree <= 12.5 and

round_Degree_mi <= 0.5 and

round_Phi > 0.12362469732761383 and

round_StdDev_Transcript_length <= 1011.6593627929688),

-0.00028572000111677241414 * (not Group == u'NDNE' and

round_Degree > 4.5 and

round_LofTool <= 0.9827499985694885 and

round_Gene_Length_bp > 39266.5),

-0.025054992712295911378 * (round_Degree <= 4.5 and

round_Degree_mi > 0.5 and

round_StdDev_Transcript_length > 1049.45654296875),

0.14453713378768545672 * (round_Closeness > 0.3149999976158142 and

2.642360210418701 < round_missense_Z <= 4.0604472160339355),

-0.019256270589407667448 * (round_StdDev_Transcript_length <= 169.24288940429688 and

round_Average_Transcript_length > 2318.75),

-0.072220477391367943198 * (Group == u'NDNE' and

round_Degree_mi <= 0.5 and

round_s_het <= 0.02767527475953102),

0.036591368423358389128 * (round_dN_dS_Chimp_mi > 0.5 and

round_missense_Z <= 3.2976694107055664 and

round_Average_Transcript_length > 2056.5712890625 and

round_Exon_Count <= 240.5),

0.0098218305890553827403 * (round_missense_Z <= 4.050085067749023 and

round_Transcript_count > 10.5 and

round_Average_Transcript_length > 2037.5650634765625),

0.0025043504719714578151 * (Group == u'CM' and

3.5 < round_Degree <= 56.5 and

round_Exon_Count <= 253.5),

-0.00027213615358005234643 * (not Group == u'MNC' and

round_dN_dS_Chimp_mi <= 0.5 and

round_Blomen_KBM7 <= -0.12487166374921799 and

round_s_het > 0.014458265155553818),

-0.012962701962375372186 * (round_Phi > 0.002197184134274721 and

round_LofTool <= 0.6634999513626099 and

round_Exon_Count > 88.5),

0.01726754044835551033 * (not Group == u'NDNE' and

round_missense_Z > 2.5613200664520264 and

round_s_het > 0.01700003445148468 and

round_Exon_Count <= 86.5),

0.047407821001623717816 * (not Group == u'CM' and

not Group == u'MNC' and

round_Blomen_KBM7 <= -0.5148604512214661 and

round_Transcript_count > 26.5),

-0.012239859477472964447 * (not Group == u'MNC' and

round_End <= 100419720.0 and

round_Degree <= 34.5 and

round_Tajima__s_D_regulatory > -1.2855000495910645),

-0.010213778329625312902 * (round_Degree <= 45.0 and

round_dN_dS_Chimp_mi > 0.5 and

round_Transcript_count > 1.5 and

round_StdDev_Transcript_length <= 1942.072509765625),

0.0026979756228996763096 * (round_Degree_mi <= 0.5 and

round_Tajima__s_D_regulatory > -1.2885000705718994 and

round_Blomen_KBM7 <= -0.2516775131225586 and

round_s_het > 0.015896810218691826),

-0.034743371535693644281 * (not Group == u'NDNE' and

round_Closeness <= 0.2549999952316284 and

round_missense_Z <= 1.43977952003479),

-0.00032670412491793654431 * (round_Degree <= 60.5 and

round_dN_dS_Chimp_mi <= 0.5 and

round_Blomen_KBM7 > -0.5153281092643738 and

round_Exon_Count <= 154.5),

-0.0069335310400958204829 * (round_Degree <= 3.5 and

round_Closeness <= 0.3149999976158142 and

round_Blomen_KBM7 <= -0.25219112634658813 and

round_LofTool <= 0.8695000410079956),

-0.084412030034841656345 * (not Group == u'NDNE' and

round_Closeness <= 0.2549999952316284 and

round_LofTool > 0.8554999828338623),

-0.0068004078113453672594 * (round_Degree > 18.5 and

round_Blomen_KBM7 <= -0.14956465363502502 and

round_LofTool > 0.659500002861023),

0.019252153925983447186 * (round_Degree > 62.5),

-0.0049752751404963432152 * (not Group == u'CM' and

round_dN_dS_Chimp_mi <= 0.5 and

round_Tajima__s_D_regulatory <= 1.062999963760376 and

round_Blomen_KBM7_mi <= 0.5),

0.003356968599162736891 * (round_Phi > 0.12447576969861984 and

round_missense_Z > 4.050085067749023),

-0.011793238037396919921 * (round_Closeness <= 0.2549999952316284 and

round_Blomen_KBM7_mi <= 0.5 and

round_s_het_mi <= 0.5 and

round_StdDev_Transcript_length <= 2625.484375),

-0.051565317012112775463 * (round_missense_Z > 4.052361965179443 and

round_LofTool <= 0.9499499797821045),

-0.019198707422328438466 * (round_End <= 100419720.0 and

round_Blomen_KBM7_mi > 0.5 and

round_Exon_Count <= 221.5),

0.028560411574742990137 * (round_Degree <= 12.5 and

round_Blomen_KBM7 <= -0.1114160418510437 and

round_StdDev_Transcript_length > 2377.90625 and

round_Average_Transcript_length > 2169.3193359375),

-0.0081602144775651253017 * (round_Degree > 3.5 and

round_Closeness <= 0.3149999976158142 and

round_LofTool > 0.6514999866485596),

0.023083051924208931871 * (round_Degree <= 12.5 and

round_dN_dS_Chimp_mi <= 0.5 and

round_Blomen_KBM7 <= -0.2521226406097412 and

round_Gene_Length_bp > 2814.5),

0.046139548753990018704 * (round_dN_dS_Chimp_mi <= 0.5 and

round_LofTool > 0.6634999513626099 and

round_Exon_Count > 162.5),

0.0082417292345994958014 * (Group == u'NDNE' and

round_Blomen_KBM7 > -0.5146430730819702 and

round_Transcript_count <= 20.5 and

round_StdDev_Transcript_length <= 2418.21435546875),

0.041904863071896121529 * (round_End > 100419720.0 and

round_dN_dS_Chimp_mi > 0.5 and

round_LofTool <= 0.994350016117096),

-0.058523347259765781669 * (not Group == u'MNC' and

167.33212280273438 < round_StdDev_Transcript_length <= 615.8639526367188),

-0.13397370099236294294 * (not Group == u'MNC' and

round_Tajima__s_D_regulatory > -0.4115000069141388 and

round_Gene_Length_bp <= 9988.0),

-0.0025281542638851562527 * (not Group == u'CM' and

not Group == u'MNC' and

round_Blomen_KBM7 <= -0.5148604512214661 and

round_Transcript_count <= 26.5),

-0.014816630169283761392 * (Group == u'NDNE' and

round_Degree <= 12.5 and

round_missense_Z <= 3.2976694107055664 and

round_Exon_Count <= 223.5),

-0.0016748509383901300038 * (round_Closeness <= 0.3149999976158142 and

round_LofTool <= 0.6634999513626099 and

round_Transcript_count <= 20.5 and

round_StdDev_Transcript_length <= 1968.24755859375),

0.021419335487036086224 * (round_Closeness > 0.2549999952316284 and

round_missense_Z <= 3.334120750427246 and

round_s_het > 0.014458265155553818),

0.053846292322283426102 * (round_Phi > 3.313508932478726e-05 and

3.2976694107055664 < round_missense_Z <= 4.052361965179443),

-0.094256185734778688556 * (not Group == u'CM' and

not Group == u'MNC' and

round_Degree <= 60.5 and

round_missense_Z <= 4.031624794006348),

0.044101606757228337119 * (round_Degree <= 35.5 and

round_dN_dS_Chimp <= 0.5950000286102295 and

round_Blomen_KBM7 <= -0.12487166374921799 and

round_missense_Z <= 4.052361965179443),

0.034744638865665013194 * (not Group == u'NDNE' and

round_Degree <= 3.5 and

round_StdDev_Transcript_length > 1049.45654296875),

-0.0015117432304791230541 * (round_End > 127552112.0 and

round_dN_dS_Chimp <= 0.5950000286102295 and

round_Tajima__s_D_regulatory <= 0.4165000021457672 and

round_missense_Z <= 4.052361965179443),

-0.0043696277889927453292 * (not Group == u'NDNE' and

0.2549999952316284 < round_Closeness <= 0.3149999976158142 and

round_Phi <= 0.8754478693008423),

0.035691787319774168075 * (not Group == u'NDNE' and

round_Closeness <= 0.3149999976158142 and

round_Phi > 0.8768634796142578),

-0.0038248717892900479035 * (not Group == u'NDNE' and

round_Degree > 3.5 and

round_Phi > 0.8768634796142578 and

round_LofTool <= 0.9829000234603882),

-3.0717810457948354157E-07 * (round_Average_Transcript_length),

0.11374755291070159924 * (round_Tajima__s_D_regulatory <= 0.4235000014305115 and

round_Blomen_KBM7 <= -0.2521226406097412 and

round_s_het <= 0.015603477135300636),

-0.0047514279836939361107 * (round_dN_dS_Chimp_mi <= 0.5 and

round_Blomen_KBM7 > -0.5181520581245422 and

round_Transcript_count <= 26.5 and

round_StdDev_Transcript_length > 169.24288940429688),

-0.012826511723195505726 * (not Group == u'NDNE' and

round_Degree <= 12.5 and

round_StdDev_Transcript_length <= 1418.164794921875),

0.016581885867794556033 * (round_Tajima__s_D_regulatory <= 1.4184999465942383 and

round_Phi > 7.604442998854211e-06 and

round_Gene_Length_bp <= 9942.0),

-0.021957726079143407433 * (not Group == u'NDNE' and

round_Closeness <= 0.2549999952316284 and

round_Phi > 0.4316735863685608),

-0.023063757621387480368 * (Group == u'CNM'),

0.0066162516266622334662 * (round_Degree <= 10.5 and

round_Degree_mi <= 0.5 and

round_Phi > 0.13315337896347046 and

round_LofTool <= 0.9311000108718872),

-0.011550473054156200695 * (round_dN_dS_Chimp > 0.5950000286102295 and

round_missense_Z_mi <= 0.5 and

round_LofTool > 0.04450000077486038),

0.020911928595311275736 * (round_Closeness <= 0.7100000381469727 and

round_dN_dS_Chimp <= 0.5950000286102295 and

round_missense_Z <= 4.031624794006348 and

round_Gene_Length_bp <= 52859.0),

0.0081602582422299618781 * (round_Degree_mi > 0.5 and

round_Average_Transcript_length > 2550.24072265625),

0.00011519157793123054349 * (round_Blomen_KBM7 <= -0.2516775131225586 and

round_missense_Z <= 3.308867931365967 and

round_s_het > 0.015855055302381516),

-0.017088815875378346454 * (round_s_het <= 0.01700003445148468 and

round_Exon_Count <= 86.5),

-0.0052479725965815775951 * (round_dN_dS_Chimp > 0.5849999785423279 and

round_Tajima__s_D_regulatory > 0.4165000021457672),

-0.0062430452918762879139 * (round_Closeness <= 0.32499998807907104 and

round_missense_Z > 2.571293354034424 and

round_Transcript_count <= 10.5),

-0.011579335820359673917 * (round_End <= 100419720.0 and

round_Tajima__s_D_regulatory > -1.2885000705718994 and

round_missense_Z_mi <= 0.5 and

round_StdDev_Transcript_length <= 2093.128662109375),

-0.021952172218480371646 * (round_missense_Z > 4.052361965179443 and

round_Exon_Count <= 77.5),

2.1100283553908253599E-08 * (round_Gene_Length_bp),

0.030085702774348684757 * (round_Closeness > 0.3149999976158142 and

round_Phi > 0.0015477617271244526 and

round_StdDev_Transcript_length <= 626.7237548828125),

-0.0047648667314961314426 * (round_Phi <= 0.00015248148702085018),

0.0082603409156458418305 * (not Group == u'CM' and

round_dN_dS_Chimp <= 0.5950000286102295 and

round_Blomen_KBM7_mi <= 0.5 and

round_missense_Z <= 4.031624794006348),

-0.073398950654784411718 * (round_End <= 100419720.0 and

0.3050000071525574 < round_Closeness <= 0.3149999976158142 and

round_dN_dS_Chimp <= 0.5950000286102295),

0.021089497152677241093 * (round_Closeness > 0.3149999976158142 and

round_Tajima__s_D_regulatory <= 0.5485000014305115 and

round_Blomen_KBM7 <= -0.25219112634658813 and

round_LofTool <= 0.6634999513626099),

0.076308296509059625468 * (round_Closeness <= 0.32499998807907104 and

round_Tajima__s_D_regulatory <= -1.2874999046325684 and

round_Blomen_KBM7 <= -0.41121259331703186),

0.030893043174464097922 * (not Group == u'CM' and

Group == u'MNC' and

round_s_het > 0.028055116534233093),

-0.035827548812507541143 * (round_Degree > 3.5 and

round_Phi <= 0.12447576969861984 and

round_Transcript_count <= 19.5 and

round_StdDev_Transcript_length > 1098.5848388671875),

-0.005052833399117633538 * (Group == u'NDNE' and

round_Degree > 3.5 and

round_Phi > 0.7055177092552185),

0.017529633814622507665 * (round_Degree > 5.5 and

round_Closeness > 0.3149999976158142 and

round_StdDev_Transcript_length > 649.8057861328125),

0.0043005984594983578603 * (round_Tajima__s_D_regulatory > 0.4104999899864197 and

round_Blomen_KBM7 <= -0.5105985403060913 and

round_s_het > 0.014667890034615993 and

round_Exon_Count <= 88.5),

-0.0067945595152844873166 * (Group == u'NDNE' and

round_Degree <= 35.5 and

round_Gene_Length_bp <= 65653.0 and

round_StdDev_Transcript_length > 590.2589111328125),

-0.0039223340038751991835 * (round_Closeness <= 0.3149999976158142 and

round_dN_dS_Chimp <= 0.5950000286102295 and

round_Blomen_KBM7 <= -0.25219112634658813 and

round_missense_Z <= 3.2974047660827637),

0.04364110373848632124 * (-1.2874999046325684 < round_Tajima__s_D_regulatory <= 1.4165000915527344 and

round_missense_Z > 4.028769493103027 and

round_Transcript_count > 20.5),

-0.0043482169941988554202 * (Group == u'NDNE' and

round_Tajima__s_D_regulatory <= 0.5570000410079956 and

round_LofTool > 0.8105000257492065),

-0.067478416068065039113 * (not Group == u'MNC' and

round_dN_dS_Chimp_mi <= 0.5 and

round_missense_Z <= 3.2976694107055664 and

round_StdDev_Transcript_length <= 987.5745849609375),

0.05856455668737312048 * (Group == u'MNC'),

0.0087343085663817605913 * (round_Tajima__s_D_regulatory > 0.4165000021457672 and

round_LofTool > 0.6634999513626099),

0.028953842017158313432 * (not Group == u'NDNE' and

round_Closeness > 0.2549999952316284 and

round_Phi <= 0.8768634796142578 and

round_LofTool > 0.9872499704360962),

-0.011814802013852038556 * (round_Closeness > 0.3149999976158142 and

round_dN_dS_Chimp_mi <= 0.5 and

round_Average_Transcript_length <= 2037.5650634765625),

0.024333688158167710719 * (round_Closeness > 0.3149999976158142 and

round_Phi > 0.12447576969861984 and

round_missense_Z <= 3.302974224090576),

-0.034348155117580804474 * (round_Degree_mi <= 0.5 and

round_Closeness <= 0.2549999952316284 and

round_Phi <= 0.8162848949432373),

0.012477065486332037866 * (round_Degree <= 4.5 and

round_missense_Z > 1.43977952003479),

-0.0055992038299544763177 * (round_dN_dS_Chimp_mi <= 0.5 and

0.6634999513626099 < round_LofTool <= 0.9922449588775635 and

round_Exon_Count <= 162.5),

0.012688866869836799844 * (Group == u'END'),

0.051122519055613512007 * (round_Degree > 70.0 and

round_Phi > 0.12447576969861984 and

round_Exon_Count <= 156.5),

0.033172549145610998045 * (not Group == u'MNC' and

round_End <= 100331008.0 and

round_missense_Z <= 3.308867931365967),

0.042438817871726271236 * (round_Blomen_KBM7 <= -0.5158457159996033 and

round_Gene_Length_bp <= 53140.5),

0.018517202101923656982 * (round_End > 100419720.0 and

round_missense_Z > 3.996763229370117),

0.0097170358540753076076 * (not Group == u'NDNE' and

round_StdDev_Transcript_length > 590.2589111328125 and

round_Exon_Count > 253.5),

0.041889875414339285131 * (Group == u'NDNE' and

round_Degree <= 3.5 and

round_Phi > 0.14934945106506348),

0.1159141151153197935 * (round_dN_dS_Chimp_mi),

-0.036397079913846719368 * (round_Degree <= 3.5 and

round_Phi <= 0.12447576969861984 and

round_Transcript_count <= 19.5 and

round_StdDev_Transcript_length > 1098.5848388671875),

-0.040646373044763282889 * (round_Closeness <= 0.2549999952316284 and

round_missense_Z <= 3.2976694107055664 and

round_StdDev_Transcript_length > 987.5745849609375),

0.0034491772056603540834 * (not Group == u'NDNE' and

round_Degree <= 62.5 and

round_Transcript_count <= 20.5 and

round_StdDev_Transcript_length > 580.6193237304688),

0.010753897831233117169 * (round_Closeness > 0.3149999976158142 and

round_Blomen_KBM7 <= -0.5146373510360718),

-0.0029093503719793034797 * (round_dN_dS_Chimp <= 0.5950000286102295 and

round_missense_Z <= 3.2976694107055664 and

round_LofTool <= 0.9922449588775635),

0.0085107244663394625989 * (round_s_het_mi),

-0.016375026470318413546 * (not Group == u'CM' and

not Group == u'MNC' and

round_End <= 121391536.0 and

round_Blomen_KBM7 <= -0.49977701902389526),

0.0064940396356499840991 * (not Group == u'NDNE' and

round_Closeness <= 0.32499998807907104 and

round_missense_Z > 1.43977952003479 and

round_Gene_Length_bp > 47610.5),

0.1894264397753082918 * (round_Degree <= 4.5 and

round_LofTool > 0.9868500232696533 and

round_StdDev_Transcript_length > 1049.45654296875),

-0.023331859677601131386 * (not Group == u'MNC' and

round_Degree <= 35.5 and

round_Tajima__s_D_regulatory > -1.2855000495910645 and

round_Gene_Length_bp <= 315852.5),

0.029766225515022122494 * (round_dN_dS_Chimp <= 0.5950000286102295 and

round_Gene_Length_bp > 343581.5),

0.16369767760948211732 * (Group == u'NDNE' and

round_Degree <= 4.5 and

round_StdDev_Transcript_length <= 714.263427734375),

-0.024442885761271002099 * (Group == u'NDNE' and

round_Degree_mi <= 0.5 and

round_StdDev_Transcript_length <= 661.5775146484375),

-0.0042331491676081359904 * (round_Closeness <= 0.3149999976158142 and

round_Phi > 0.12447576969861984 and

round_LofTool > 0.8825000524520874 and

round_Gene_Length_bp <= 54522.0),

0.0376150466960103666 * (round_Tajima__s_D_regulatory <= 0.4235000014305115 and

round_LofTool > 0.9922800064086914),

0.030185637919566413873 * (not Group == u'NDNE' and

round_Degree_mi <= 0.5 and

round_Closeness <= 0.3149999976158142 and

round_Average_Transcript_length > 2570.535888671875),

0.0075692806955283938389 * (round_Closeness <= 0.3149999976158142 and

round_Phi > 0.12447576969861984 and

round_LofTool <= 0.9910449981689453 and

round_Gene_Length_bp > 54522.0),

0.093970991187771663045 * (round_Closeness),

0.0051682327079576413295 * (round_Blomen_KBM7 > -0.5153281092643738 and

0.49326902627944946 < round_missense_Z <= 4.031624794006348 and

round_Gene_Length_bp <= 53047.5),

-0.00063682044972014964761 * (not Group == u'CM' and

not Group == u'MNC' and

round_End > 121391536.0 and

round_Blomen_KBM7 <= -0.49977701902389526),

0.1305902319414564694 * (not Group == u'NDNE' and

round_StdDev_Transcript_length > 636.7213134765625 and

round_Exon_Count > 253.5),

-0.028105123991677912615 * (round_dN_dS_Chimp_mi <= 0.5 and

round_Blomen_KBM7 > -0.5146373510360718 and

round_LofTool <= 0.9922449588775635 and

round_Transcript_count > 20.5),

-0.071733384292753485378 * (round_dN_dS_Chimp_mi <= 0.5 and

round_Blomen_KBM7 > -0.5146373510360718 and

round_Transcript_count <= 20.5 and

round_StdDev_Transcript_length <= 2388.5126953125),

-0.0014944870184999594492 * (round_Closeness > 0.3149999976158142 and

round_missense_Z <= 2.571293354034424 and

round_StdDev_Transcript_length > 1450.0491943359375),

-0.037305572747207686735 * (round_Degree <= 12.5 and

round_missense_Z > 4.052361965179443 and

round_Gene_Length_bp > 2814.5 and

round_Exon_Count <= 413.0),

-0.013677846268730356125 * (round_Closeness <= 0.3449999988079071 and

3.2976694107055664 < round_missense_Z <= 4.052361965179443 and

round_Average_Transcript_length <= 2037.5650634765625),

-0.011008296654006112167 * (round_Degree <= 3.5 and

round_Phi > 0.0009046811610460281 and

round_LofTool <= 0.6634999513626099),

-0.066509812215818378545 * (round_Degree <= 12.5 and

round_dN_dS_Chimp_mi <= 0.5 and

0.5915000438690186 < round_LofTool <= 0.6634999513626099),

0.043755311507365633739 * (round_dN_dS_Chimp_mi <= 0.5 and

round_missense_Z <= 3.2976694107055664 and

round_Average_Transcript_length > 2056.5712890625 and

round_Exon_Count <= 240.5),

0.01113235474873714749 * (round_Degree <= 60.5 and

round_dN_dS_Chimp_mi <= 0.5 and

round_LofTool <= 0.6634999513626099 and

round_Transcript_count > 20.5),

0.029791550463155375139 * (round_End > 100419720.0 and

round_dN_dS_Chimp_mi <= 0.5 and

round_LofTool <= 0.994350016117096),

-0.0065287331094041282237 * (round_Degree <= 60.0 and

round_Phi <= 0.12447576969861984 and

round_Exon_Count > 83.5),

-0.0045345154906442954046 * (round_Tajima__s_D_regulatory > -1.2874999046325684 and

round_missense_Z <= 3.2976694107055664 and

round_missense_Z_mi <= 0.5 and

round_LofTool > 0.6634999513626099),

-0.0042090971907015710396 * (round_Closeness <= 0.2549999952316284 and

round_Phi > 0.0015477617271244526 and

round_StdDev_Transcript_length <= 2427.245361328125 and

round_Exon_Count <= 96.5),

0.0064827603925068496768 * (round_Degree <= 10.5 and

round_LofTool <= 0.659500002861023 and

round_Gene_Length_bp > 37942.5),

0.090064277946272239261 * (round_Degree > 3.5 and

round_Closeness <= 0.3149999976158142 and

round_Gene_Length_bp <= 41501.0),

-0.016228231105145558139 * (round_Degree_mi > 0.5 and

round_StdDev_Transcript_length <= 613.3181762695312),

-0.024541945681890484088 * (round_s_het <= 0.025150161236524582 and

round_Transcript_count <= 13.5 and

round_Average_Transcript_length > 1873.0999755859375),

0.088655920423249531814 * (round_End <= 110010712.0 and

round_Degree <= 61.5 and

round_Degree_mi <= 0.5 and

round_missense_Z <= 4.052361965179443),

0.033084801199255609028 * (Group == u'NDNE' and

round_Degree <= 12.5 and

round_s_het > 0.02518850564956665),

0.01835275341503877361 * (round_Degree > 12.5 and

round_Degree_mi <= 0.5 and

round_Closeness > 0.3149999976158142 and

round_StdDev_Transcript_length > 1285.35791015625),

-0.041044206485432332965 * (Group == u'NDNE' and

round_Degree <= 4.5 and

round_StdDev_Transcript_length <= 1049.45654296875),

-0.039696369932485681131 * (round_Phi > 0.998741626739502 and

round_Gene_Length_bp > 9906.5 and

round_StdDev_Transcript_length > 166.57864379882812 and

round_Average_Transcript_length > 1956.6083984375),

-0.012496735141249921616 * (round_dN_dS_Chimp <= 0.5950000286102295 and

round_missense_Z > 4.052361965179443 and

round_Transcript_count <= 10.5 and

round_Average_Transcript_length <= 6597.4873046875),

0.039674947245171322818 * (not Group == u'NDNE' and

round_Closeness <= 0.3149999976158142 and

round_Phi > 0.12447576969861984 and

round_Gene_Length_bp <= 45328.0),

-0.30567366547201163529 * (round_Degree <= 4.5 and

round_Degree_mi <= 0.5 and

round_StdDev_Transcript_length > 1049.45654296875) ])

def get_type_conversion():

return {

u'Tajima\'s D regulatory': {'convert_func': parse_nonstandard_na, 'convert_args': None},

u'Degree': {'convert_func': parse_nonstandard_na, 'convert_args': None},

u'dN/dS Chimp': {'convert_func': parse_nonstandard_na, 'convert_args': None},

u'Closeness': {'convert_func': parse_nonstandard_na, 'convert_args': None},}

INDICATOR_COLS = [u'Blomen KBM7', u'Degree', u'LofTool', u'Phi', u'Tajima\'s D regulatory', u'dN/dS Chimp', u'missense_Z', u's_het']

IMPUTE_VALUES = {

u'Average Transcript length': 1932.128571,

u'Blomen KBM7': -0.499215,

u'Closeness': 0.250000,

u'Degree': 3.000000,

u'End': 58139967.000000,

u'Exon Count': 36.000000,

u'Gene Length bp': 28439.000000,

u'LofTool': 0.502000,

u'Phi': 0.002401,

u'StdDev Transcript length': 899.036969,

u'Tajima\'s D regulatory': 0.143000,

u'Transcript count': 6.000000,

u'dN/dS Chimp': 0.230000,

u'missense_Z': 0.493769,

u's_het': 0.017813,}

def bag_of_words(text):

""" set of whole words in a block of text """

if type(text) == float:

return set()

return set(word.lower() for word in

re.findall(r'\w+', text, re.UNICODE | re.IGNORECASE))

def parse_date(x, date_format):

""" convert date strings to numeric values. """

try:

# float values no longer pass isinstance(x, np.float64)

if isinstance(x, (np.float64, float)):

x = long_type(x)

if '%f' in date_format and date_format.startswith('v2'):

temp = str(x)

if re.search('[\+-][0-9]+$', temp):

temp = re.sub('[\+-][0-9]+$', '', temp)

date_format = date_format[2:]

dt = datetime.strptime(temp, date_format)

sec = calendar.timegm(dt.timetuple())

return sec * 1000 + dt.microsecond // 1000

elif '%M' in date_format:

temp = str(x)

if re.search('[\+-][0-9]+$', temp):

temp = re.sub('[\+-][0-9]+$', '', temp)

return calendar.timegm(datetime.strptime(temp, date_format).timetuple())

else:

return datetime.strptime(str(x), date_format).toordinal()

except:

return float('nan')

def parse_percentage(s):

""" remove percent sign so percentage variables can be converted to numeric """

if isinstance(s, float):

return s

if isinstance(s, int):

return float(s)

try:

return float(s.replace('%', ''))

except:

return float('nan')

def parse_nonstandard_na(s):

""" if a column contains numbers and a unique non-numeric,

then the non-numeric is considered to be N/A

"""

try:

ret = float(s)

if np.isinf(ret):

return float('nan')

return ret

except:

return float('nan')

def parse_length(s):

""" convert feet and inches as string to inches as numeric """

try:

if '"' in s and "'" in s:

sp = s.split("'")

return float(sp[0]) * 12 + float(sp[1].replace('"', ''))

else:

if "'" in s:

return float(s.replace("'", '')) * 12

else:

return float(s.replace('"', ''))

except:

return float('nan')

def parse_currency(s):

""" strip currency characters and commas from currency columns """

if not isinstance(s, text_type):

return float('nan')

s = re.sub(u'[\$\u20AC\u00A3\uFFE1\u00A5\uFFE5]|(EUR)', '', s)

s = s.replace(',', '')

try:

return float(s)

except:

return float('nan')

def parse_currency_replace_cents_period(val, currency_symbol):

try:

if np.isnan(val):

return val

except TypeError:

pass

if not isinstance(val, string_types):

raise ValueError('Found wrong value for currency: {}'.format(val))

try:

val = val.replace(currency_symbol, "", 1)

val = val.replace(" ", "")

val = val.replace(",", "")

val = float(val)

except ValueError:

val = float('nan')

return val

def parse_currency_replace_cents_comma(val, currency_symbol):

try:

if np.isnan(val):

return val

except TypeError:

pass

if not isinstance(val, string_types):

raise ValueError('Found wrong value for currency: {}'.format(val))

try:

val = val.replace(currency_symbol, "", 1)

val = val.replace(" ", "")

val = val.replace(".", "")

val = val.replace(",", ".")

val = float(val)

except ValueError:

val = float('nan')

return val

def parse_currency_replace_no_cents(val, currency_symbol):

try:

if np.isnan(val):

return val

except TypeError:

pass

if not isinstance(val, string_types):

raise ValueError('Found wrong value for currency: {}'.format(val))

try:

val = val.replace(currency_symbol, "", 1)

val = val.replace(" ", "")

val = val.replace(",", "")

val = val.replace(".", "")

val = float(val)

except ValueError:

val = float('nan')

return val

def parse_numeric_types(ds):

""" convert strings with numeric types (date, currency, etc.)

to actual numeric values """

TYPE_CONVERSION = get_type_conversion()

for col in ds.columns:

if col in TYPE_CONVERSION:

convert_func = TYPE_CONVERSION[col]['convert_func']

convert_args = TYPE_CONVERSION[col]['convert_args']

ds[col] = ds[col].apply(convert_func, args=convert_args)

return ds

def sanitize_name(name):

safe = name.strip().replace("-", "_").replace("$", "_").replace(".", "_")

safe = safe.replace("{", "_").replace("}", "_")

safe = safe.replace('"', '_')

safe = safe.replace("\n", "_")

safe = safe.replace("\r", "_")

return safe

def rename_columns(ds):

new_names = {}

existing_names = set()

blank_index = 0

for old_col in ds.columns:

col = sanitize_name(old_col)

if col == '':

col = 'Unnamed: %d' % blank_index

blank_index += 1

if col in existing_names:

raise ValueError('Duplication detected. Column with name=['

+ old_col + '] was preprocessed to['

+ col + '] that already exists')

existing_names.add(col)

new_names[old_col] = col

ds.rename(columns=new_names, inplace=True)

return ds

def add_missing_indicators(ds):

for col in INDICATOR_COLS:

ds[col + '-mi'] = ds[col].isnull().astype(int)

return ds

def impute_values(ds):

for col in ds:

if col in IMPUTE_VALUES:

ds.loc[ds[col].isnull(), col] = IMPUTE_VALUES[col]

return ds

BIG_LEVELS = {

u'Group': [

u'CM',

u'CNM',

u'END',

u'MNC',

u'NDNE',

],

}

SMALL_NULLS = {

u'Group': 1,

}

VAR_TYPES = {

u'Average Transcript length': 'N',

u'Blomen KBM7': 'N',

u'Closeness': 'N',

u'Degree': 'N',

u'End': 'N',

u'Exon Count': 'N',

u'Gene Length bp': 'N',

u'Group': 'C',

u'LofTool': 'N',

u'Phi': 'N',

u'StdDev Transcript length': 'N',

u'Tajima\'s D regulatory': 'N',

u'Transcript count': 'N',

u'dN/dS Chimp': 'N',

u'missense_Z': 'N',

u's_het': 'N',

}

def combine_small_levels(ds):

for col in ds:

if BIG_LEVELS.get(col, None) is not None:

mask = np.logical_and(~ds[col].isin(BIG_LEVELS[col]), ds[col].notnull())

if np.any(mask):

ds.loc[mask, col] = 'small_count'

if SMALL_NULLS.get(col):

mask = ds[col].isnull()

if np.any(mask):

ds.loc[mask, col] = 'small_count'

if VAR_TYPES.get(col) == 'C' or VAR_TYPES.get(col) == 'T':

mask = ds[col].isnull()

if np.any(mask):

if ds[col].dtype == float:

ds[col] = ds[col].astype(object)

ds.loc[mask, col] = 'nan'

return ds

# N/A strings in addition to the ones used by Pandas read_csv()

NA_VALUES = ['null', 'na', 'n/a', '#N/A', 'N/A', '?', '.', '', 'Inf', 'INF', 'inf', '-inf', '-Inf', '-INF', ' ', 'None', 'NaN', '-nan', 'NULL', 'NA', '-1.#IND', '1.#IND', '-1.#QNAN', '1.#QNAN', '#NA', '#N/A N/A', '-NaN', 'nan']

# True/False strings in addition to the ones used by Pandas read_csv()

TRUE_VALUES = ['TRUE', 'True', 'true']

FALSE_VALUES = ['FALSE', 'False', 'false']

DEFAULT_ENCODING = 'utf8'

REQUIRED_COLUMNS = [u"Average Transcript length",u"Blomen KBM7",u"Closeness",u"Degree",u"End",u"Exon Count",u"Gene Length bp",u"Group",u"LofTool",u"Phi",u"StdDev Transcript length",u"Tajima's D regulatory",u"Transcript count",u"dN/dS Chimp",u"missense_Z",u"s_het"]

def validate_columns(column_list):

if set(REQUIRED_COLUMNS) <= set(column_list):

return True

else :

raise ValueError("Required columns missing: %s" %

(set(REQUIRED_COLUMNS) - set(column_list)))

def convert_bool(ds):

TYPE_CONVERSION = get_type_conversion()

for col in ds.columns:

if VAR_TYPES.get(col) == 'C' and ds[col].dtype in (int, float):

mask = ds[col].notnull()

ds[col] = ds[col].astype(object)

ds.loc[mask, col] = ds.loc[mask, col].astype(text_type)

elif VAR_TYPES.get(col) == 'N' and ds[col].dtype == bool:

ds[col] = ds[col].astype(float)

elif ds[col].dtype == bool:

ds[col] = ds[col].astype(text_type)

elif ds[col].dtype == object:

if VAR_TYPES.get(col) == 'N' and col not in TYPE_CONVERSION:

mask = ds[col].apply(lambda x: x in TRUE_VALUES)

if np.any(mask):

ds.loc[mask, col] = 1

mask = ds[col].apply(lambda x: x in FALSE_VALUES)

if np.any(mask):

ds.loc[mask, col] = 0

ds[col] = ds[col].astype(float)

elif TYPE_CONVERSION.get(col) is None:

mask = ds[col].notnull()

ds.loc[mask, col] = ds.loc[mask, col].astype(text_type)

return ds

def get_dtypes():

return {a: object for a, b in VAR_TYPES.items() if b == 'C'}

def predict_dataframe(ds):

return ds.apply(predict, axis=1)

def run_dataframe(ds):

ds = rename_columns(ds)

ds = convert_bool(ds)

validate_columns(ds.columns)

ds = parse_numeric_types(ds)

ds = add_missing_indicators(ds)

ds = impute_values(ds)

ds = combine_small_levels(ds)

prediction = 1/(1 + np.exp(-predict_dataframe(ds)))

return prediction

def run(dataset_path, output_path, encoding=None):

if encoding is None:

encoding = DEFAULT_ENCODING

ds = pd.read_csv(dataset_path, na_values=NA_VALUES, low_memory=False,

dtype=get_dtypes(), encoding=encoding)

prediction = run_dataframe(ds)

prediction_file = output_path

prediction.name = 'Prediction'

prediction.to_csv(prediction_file, header=True, index_label='Index')

def _construct_parser():

import argparse

parser = argparse.ArgumentParser(description='Make offline predictions with DataRobot Prime')

parser.add_argument(

'--encoding',

type=str,

help=('the encoding of the dataset you are going to make predictions with. '

'DataRobot Prime defaults to UTF-8 if not otherwise specified. See the '

'"Codecs" column of the Python-supported standards chart '

'(https://docs.python.org/2/library/codecs.html#standard-encodings) '

'for possible alternative entries.'),

metavar='<encoding>'

)

parser.add_argument(

'input_path',

type=str,

help=('a .csv file (your dataset); columns must correspond to the '

'feature set used to generate the DataRobot Prime model.'),

metavar='<data_file>'

)

parser.add_argument(

'output_path',

type=str,

help='the filename where DataRobot writes the results.',

metavar='<output_file>'

)

return parser

def _parse_command(args):

parser = _construct_parser()

parsed_args = parser.parse_args(args[1:])

if parsed_args.encoding is None:

sys.stderr.write('Warning: For input data encodings other than UTF-8, '

'search "Prime examples" in the DataRobot Users Guide at https://app.datarobot.com/docs/users-guide/index.html')

parsed_args.encoding = DEFAULT_ENCODING

return parsed_args

if __name__ == '__main__':

args = _parse_command(sys.argv)

run(args.input_path, args.output_path, encoding=args.encoding)
